# Stages of Mechanochemical Depolymerization of Poly(styrene) Powder in Oxidative and Inert Atmospheres

**DOI:** 10.1021/acssuschemeng.5c05942

**Published:** 2025-10-26

**Authors:** Yuchen Chang, Adrian H. Hergesell, Claire L. Seitzinger, Aubrey M. Hepstall, Ina Vollmer, Carsten Sievers

**Affiliations:** † School of Chemical & Biomolecular Engineering, 1372 Georgia Institute of Technology, Atlanta, Georgia 30332, United States; ‡ Inorganic Chemistry and Catalysis, Institute for Sustainable and Circular Chemistry, 8125 Utrecht University, Universiteitsweg 99, 3584 CG Utrecht, The Netherlands

**Keywords:** ball mill, mechanical grinding, radical, solid-state chemistry, polyolefin upcycling

## Abstract

Mechanochemical depolymerization of poly­(styrene) can yield its monomer styrene, but insufficient kinetic and mechanistic insight hamper productivity and efficient reactor design. Herein, ball milling of poly­(styrene) powder in a continuous flow reactor is coupled with real-time measurement of the rate of formation of individual products using in-line gas chromatography, complemented by multitechnique characterization of poly­(styrene) residue at specific time points including electron spin resonance, size exclusion chromatography, nuclear magnetic resonance spectroscopy and thermogravimetric analyses. Using this approach, three distinct stages in the depolymerization reactions are revealed, characterized by successive dominance of surface creation, surface friction and finally a molecular weight limit as the determining factor in the rate of styrene production. By comparing instantaneous product formation rates and trends in radical abundance under air and nitrogen atmospheres, oxygen is shown to be a promoter of depolymerization reactions through oxidation of polymer radical intermediates as well as a suppressor of radical migration. Minor coke formation is due to radical transfer reactions. Although conversion is nonquantitative, our study provides detailed mechanistic insights to overcome kinetic bottlenecks.

## Graphical abstract

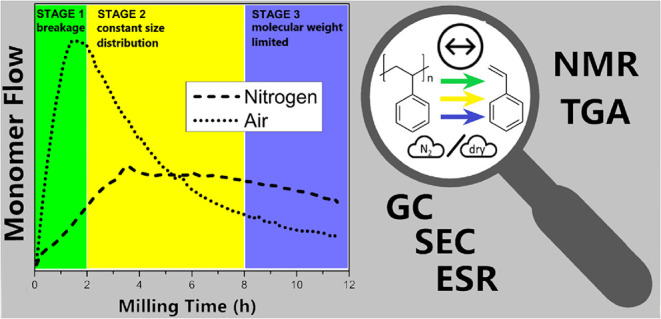

## Introduction

Plastic waste is a foremost and growing issue globally in the context of circular economy initiatives for commodity polymers.^1^ In recent years, interest has emerged in the use of trituration mechanochemistry as a tool for the chemical recycling of plastic waste.^2,3^ Mechanochemistry is advantageous because it can process polymers directly in the solid state, producing monomers through depolymerization or other small molecules by degradation. A notable example is the mechanochemical depolymerization of the commodity polyester poly­(ethylene terephthalate) at ambient conditions in a shaker ball mill reactor using alkali salt,^4^ which can swiftly achieve full conversion to monomers in 10 min at the gram scale.^5^ Successes in the mechanochemical approach have also been demonstrated for poly­(lactic acid) and polycarbonate via (solvent-free) methanolysis in ball mills.^6^ More broadly, ball milling processes at ambient conditions can advance the electrification of the chemical industry, and they are also amenable to directly utilize renewable sources of mechanical energy, such as wind or hydropower, bypassing the necessity for electrical or thermal energy conversion as intermediary.^7^ Overall, the superior green metrics of mechanochemistry^8^ make it an ideal approach for the chemical recycling of large-volume solid-state waste streams comprising just a few commodity polymers.^9,10^

The largest share of plastic waste production are olefinic polymers with hydrocarbon backbones.^10^ These polymers do not contain labile chemical bonds susceptible to attack by reagents. This typically leads to unselective reactions at high temperatures needed for, e.g., pyrolysis, and results in product mixtures which are difficult and energy intensive to separate. In contrast, ball mill grinding of poly­(styrene) (PS),^11,12^ poly­(α-methylstyrene),^13^ poly­(ethylene) (PE) and poly­(propylene)^14^ (PP) as well as various poly­(methacrylate)­s^15^ have been reported to generate their corresponding monomers in low to moderate yields, and with better selectivity to the monomer in the first two cases compared to pyrolysis conditions. The catalytic degradation of PE, PP, and other commodity olefinic plastics by ball mill grinding has also appeared in the literature. These approaches include cogrinding plastics with catalyst powder and reagents^16–19^ and chemically functionalizing the grinding surfaces of the ball mill to have catalytic activity.^20^

The common structural motif of all olefinic polymers is their backbone made up solely of C–C bonds and the fundamental mechanism of depolymerization to their respective monomers is the same. For this reason, a detailed understanding of the kinetics associated with mechanochemical depolymerization of one olefinic polymer can provide valuable insight that can be extrapolated to other types of poly­(olefins). This is especially relevant since the conversion of olefinic plastics commonly suffers from low conversion levels, and the identification of kinetic bottlenecks is imperative to increase space–time yields and increase the technological level of mechanochemical recycling. The influence of reactor operating variables such as milling frequency and catalyst loading on styrene yields from grinding PS powder in a shaker ball mill was studied extensively by Chang et al.^12^ Similar work had been carried out by Hergesell et al.^14^ on PE and PP. The thermodynamic properties of the basic depropagation reaction in inert atmosphere had also been analyzed for PS, PP and PE.^21^ To complement these insights, it would also be desirable to carry out a thorough investigation on the molecular-scale kinetics of depolymerization in the mechanochemical environment. This would address the observed role of oxygen in enhancing the rate of monomer production (from PS), the evolution of the concentration of free radicals (the key reaction intermediate in mechanochemical depolymerization) with milling time, and the interplay between molecular weight (MW) degradation and depolymerization.

In this work, grinding of PS powder in a shaker mill reactor with purge gas flow coupled with real-time downstream product analysis using gas chromatography (GC) is leveraged to elucidate the time evolution of PS depolymerization products. Electron spin resonance (ESR) spectroscopy and size-exclusion chromatography (SEC) are used alongside measurements of product formation rates to establish that mechanochemical PS depolymerization is governed by three distinct kinetic regimes. In order of occurrence, the regimes are characterized by depolymerization from surface creation at short milling times, from friction between polymer particles and between particle and grinding surfaces at intermediate milling times, and finally depolymerization that is limited by a characteristic molecular weight at long milling times. Nuclear magnetic resonance (NMR) spectroscopy and thermogravimetric analysis (TGA) offer compelling evidence for the formation of coke during mechanochemical depolymerization, consistent with the observation of more hydrogen-rich compounds among the volatile byproducts. These findings provide valuable insights into engineering approaches to mechanochemical recycling of olefinic polymers, thereby advancing commodity polymers toward circularity and sustainability.

## Materials and Methods

### Materials

PS pellets with a reported weight-average molecular weight (*M*_w_) of 192,000 g/mol were purchased from Sigma-Aldrich. Pellets were crushed to a coarse powder using a Retsch PM200 planetary mill. 50 g of PS and six 20 mm diameter steel balls were loaded into an upright cylindrical 125 mL steel grinding jar and milled for 8 min. The crushed PS grains were passed through sieves and the fraction retained between 16 and 60 mesh (250–1180 μm) was used in depolymerization experiments. This pretreatment of PS did not significantly affect the MW distribution of the material (see SI Figure S1a). Reagents used in preparation of samples for characterization, such as methanol (≥99.9%), decane (≥99%) and chloroform-d (99.9 atom %) were purchased from Sigma-Aldrich and used without further purification.

### Ball Milling Experiments

An oven-dried 25 mL custom-built 316 grade stainless steel jar with a pill-shaped interior volume (13 mm cross section radius and 30 mm straight section length) was charged with eight stainless steel grinding spheres 10 mm in diameter and 1 g of precrushed PS powder having measured number-average MW (*M_n_*) ≈ 86,000 g/mol and dispersity 2.49 (see Table S1, SI), mounted onto a Retsch MM500 Vario mixer mill, connected to PP tubing via a pair of 1/8″ Swagelok unions welded to the cylindrical face of the jar, and milled at a frequency of 30 Hz for a set amount of time. The purge gas flow rate was 60 mL/min of pure nitrogen (N_2_) or a synthetic air mixture consisting of 80% N_2_ and 20% oxygen (O_2_). The reactor exterior temperature was kept below 50 °C to avoid temperature effects on the mechanical behavior of PS particles which could confound depolymerization reaction kinetics.^12^

Products generated inside the jar during grinding were carried by the purge gas to an in-line gas chromatograph (GC, manufactured by Global Analyzer Solutions) downstream equipped with a 2 m × 0.32 mm Rtx-1, 3.0u and a 3 m × 0.32 mm Carboxen1010 column in series with a thermal conductivity detector for quantification of N_2_, and three parallel columns for the separation of C_1–3_ (3 m × 0.32 mm Rtx-1 3u and 15 m × 0.32 mm Al_2_O_3_/Na_2_SO_4_ columns), C_4–7_ (2 m × 0.28 mm MXT-1 1u and 14 m × 0.28 MXT-1 1u columns), and C_5–10_ (2 m × 0.28 mm MXT-1 0.5u and 15 m × 0.28 mm MXT-1 0.5u columns) hydrocarbon products, each equipped with its own flame ionization detector (FID) for product quantification, with identity of products determined by matching elution time to those of known pure compounds. GC sampling injections occurred once every 1.05 min on the N_2_ channel, 2.1 min on the C_1–3_ channel and 4.2 min on the other two hydrocarbon channels. For the *i*th injection, the constant purge gas flow rate *F*_0_ = 60 mL/min was used as an internal standard to counter changes in total flow *F*_tot,*i*_ due to formation of depolymerization products, with *F*_tot,*i*_ calculated by comparing the N_2_ peak area (*A*_N_2_,*i*_) of the *i*th injection with the average from three N_2_ blank injections (*A*_N_2_,b1_, *A*_N_2_,b2_, *A*_N_2_,b3_) preceding the start of milling. Molar flow *F*_P,*i*_ of each hydrocarbon product P detected in the *i*th injection is then calculated from its peak area *A*_P,*i*_ according to

1 $$F_{P,i}=F_{0}\left(\frac{A_{N_{2},b1}+A_{N_{2},b2}+A_{N_{2},b3}}{3A_{N_{2},i}}\right)\cdot \frac{1}{y_{N_{2},0}}\cdot \left(\frac{A_{P,i}}{K_{P}}\right)$$

where (*y*_N_2_,0_) is the N_2_ molar fraction in pure purge gas and *K*_P_ is a calibration factor determined by calibration of the FIDs with a mixture of methane, ethane, propane, butane, hexane, and heptane. Instantaneous product flows were integrated over time to obtain cumulative product yields. These procedures are adapted from previous work.^20^

### Characterization

Molecular weight distributions (MWDs) of solid residues recovered after 60, 120, 240, 480, and 720 min of milling were obtained using size-exclusion chromatography (SEC) performed on a Tosoh EcoSEC HLC-8320GPC equipped with TSKgel SuperMultipore HZ-M column, internal refractive index detector (RID) and Wyatt Technology DAWN8+ dynamic light scattering detector (DLS), operating at 40 °C. The eluent was chloroform containing 0.3% triethylamine at a flow rate of 0.45 mL/min and samples were prepared at concentrations of 5 to 10 mg/mL in the eluent. Electron spin resonance (ESR) spectroscopy was conducted on residues recovered from the same conditions (excluding 720 min). ESR analysis was performed on a Bruker EMXplus instrument at an X-band microwave frequency of 9.4 GHz typically using a modulation amplitude of 1 G and a modulation frequency of 100 kHz, at ambient temperature.

^13^C NMR spectroscopy experiments were conducted for the samples milled for 12 h under N_2_ and air using a Bruker AV3 400 MHz NMR spectrometer. Solid residue was collected from the ball mill at the end of a reaction, washed with 15 mL of methanol and dried in a fume hood for 2 days. 150 mg of dried solid residue was dissolved in 1.5 mL of chloroform-d (CDCl_3_) containing 0.03% (v/v) tetramethylsilane (TMS) and passed through two 0.2 μm PTFE syringe filters. Thermogravimetric analysis (TGA) experiments were also conducted on these same residues using a PerkinElmer TGA 8000 with alumina crucibles. Degradation curves were obtained using about 3–6 mg of residue at a flow rate of 45 mL/min of either N_2_ or O_2_, with a temperature program starting at 50 °C and ramping up to 600 °C at a heating rate of 10 °C/min (for N_2_) or 1000 °C at a heating rate of 20 °C/min (for O_2_).

## Results

The reaction conditions and products obtained by the grinding of PS in a shaker ball mill are depicted in Figure 1. During milling, PS particles are crushed into a fine powder. This can be seen in microscopy images of PS residue taken at 60, 120, 240, and 480 min of milling (see Figure S12, SI), which show grains 2–70 μm in size at 60 min (reduced from an average initial size above 100 μm) that become reduced to narrower distributions averaging 13 ± 8 μm by 120 min and remain at that size thereafter (see SI, Figure S13 for size distributions and S14a for average size).

**Figure 1:**
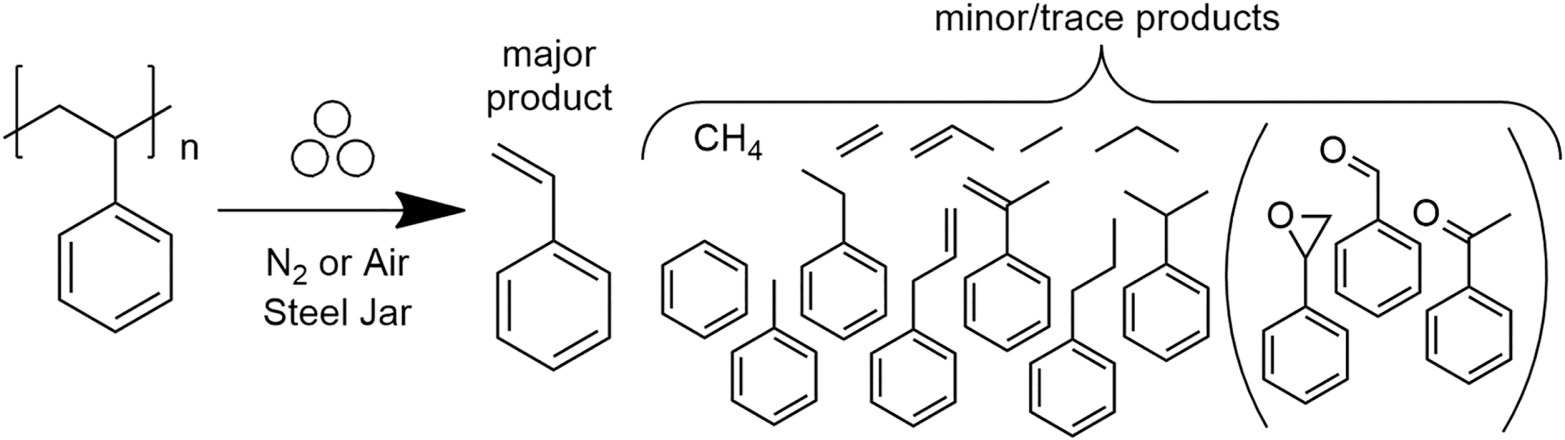
Reaction scheme of mechanochemical PS depolymerization. Aromatic products enclosed in brackets were only observed in the presence of oxygen (O_2_) from milling in dry air atmosphere.

The in-line GC analysis of the effluent stream revealed the formation of styrene, benzene and small quantities of C_1_–C_3_ hydrocarbon gases (methane, ethene, propene, trace amounts of ethane, propane) alongside previously reported^12^ volatile products. The yields of styrene and light gases for experiments conducted with flowing N_2_ (dashed lines) and air (dotted lines) atmospheres are depicted in Figure 2a,b, respectively giving 6.5 and 8.3% conversions to styrene over 720 min of continuous milling (see Table S2, SI). Figure 2a shows that the styrene yield under air was more than twice as high as under N_2_ for the first 240 min of milling but slowed down subsequently. The yields of C_1_–C_3_ hydrocarbon gases appeared unaffected by the presence of O_2_ in the purge gas until about 210 min, when yields under air gradually tapered off while yields under N_2_ continued unabated.

**Figure 2:**
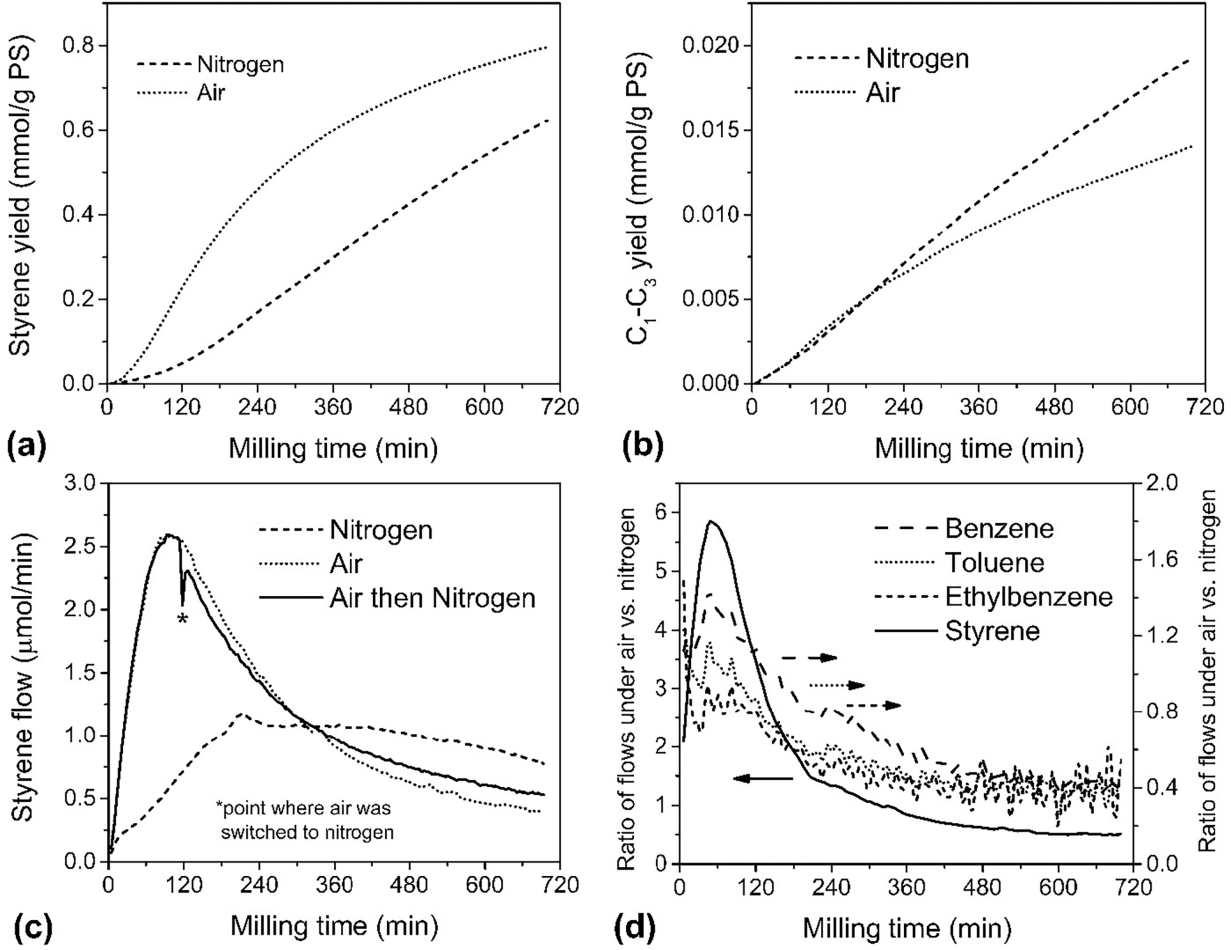
Depolymerization of 1 g of PS inside a 25 mL stainless steel reactor with eight 10 mm stainless steel balls under a continuous purge gas flow rate of 60 mL/min. Cumulative yields of styrene (a) and C_1_–C_3_ light gas (b) versus time for experiments under flowing N_2_ and air atmosphere; (c) styrene flow rate in effluent stream versus time for experiments under N_2_, air, and air switched to N_2_ at the 2 h mark (denoted by asterisk); (d) ratio of instantaneous yield under air to that under N_2_ for the four most prominent aromatic hydrocarbon products.

In-line GC analysis also allowed for the direct measurement of styrene flow (equivalent to rate of monomer production) with time, which is depicted in Figure 2c for milling under N_2_ (dashed line) and under air (dotted line). These results reveal finer details in the depolymerization kinetics that cannot be discerned from the styrene yield curve alone. Under N_2_, the styrene flow increased linearly for about the first 210 min of grinding and plateaued at 1 μmol/min of styrene. This rate was sustained for the next 270 min before the onset of a slow linear decline around 480 min of grinding. By contrast, the styrene flow under air increased much more rapidly at the start, peaking at around 120 min at under 3 μmol/min, before undergoing a more precipitous decline which only became apparent by 320 min in the corresponding yield curve in Figure 2a. This rate fell below the styrene flow under N_2_ after 320 min, although beyond 480 min the slope of the decline appeared to have settled to the same trend as the decline under N_2_. This is supported by the ratios of flows for four of the aromatic products under the two different atmospheres all approaching a nearly constant value (Figure 2d). Instantaneous yields of products depicted in Figure 1–which are the flow multiplied by sampling period of 4.2 min–are provided in the SI, Figure S6a-i. In particular, it can be seen from Figure S6i that despite styrene flow peaking at 210 versus 120 min under N_2_ and air respectively, flows of aromatics and C_1_–C_3_ hydrocarbons had all peaked by 120 min.

To determine whether sustained exposure to oxygen during milling causes permanent chemical changes to PS that would affect the depolymerization kinetics, PS was ball milled under air for 2 h before switching over to N_2_ (Figure 2c, solid line). The timing of the switch to N_2_ atmosphere was chosen to occur immediately following the maximum in styrene flow under air. The sharp dip of the measured styrene flow about 8 min after the switch was an artifact due to pressure fluctuations in the system. Despite the switch to an inert purge gas, the subsequent evolution of the flow rate continued to resemble the curve for milling under air. Beyond 480 min, the flow rate curve for this experiment also paralleled the curve for milling in N_2_ and under air.

The selectivities of depolymerization products with time are shown in Figure 3a–f. We calculated selectivities via two different methods to understand the formation of different products: (i) We referenced the formation of aromatic products against phenyl groups of PS with one phenyl group counting as one unit of product. This neglects the formation of C_1_–C_3_ gases. (ii) We referenced the formation of products against carbon atoms in the PS backbone where methane and toluene count for one unit of product; ethane, ethene, ethylbenzene and styrene count for two; benzene counts for zero, etc. Under N_2_, the selectivity of styrene calculated with respect to the pendant phenyl group (Figure 3b) increased rapidly in the first 210 min of milling and then more gradually over the next 4 h to eventually reach a steady-state value of 85 mol % at the expense of benzene, toluene and ethylbenzene. The same conclusion is reached from selectivities calculated with respect to backbone carbon atoms (Figure 3a).

**Figure 3:**
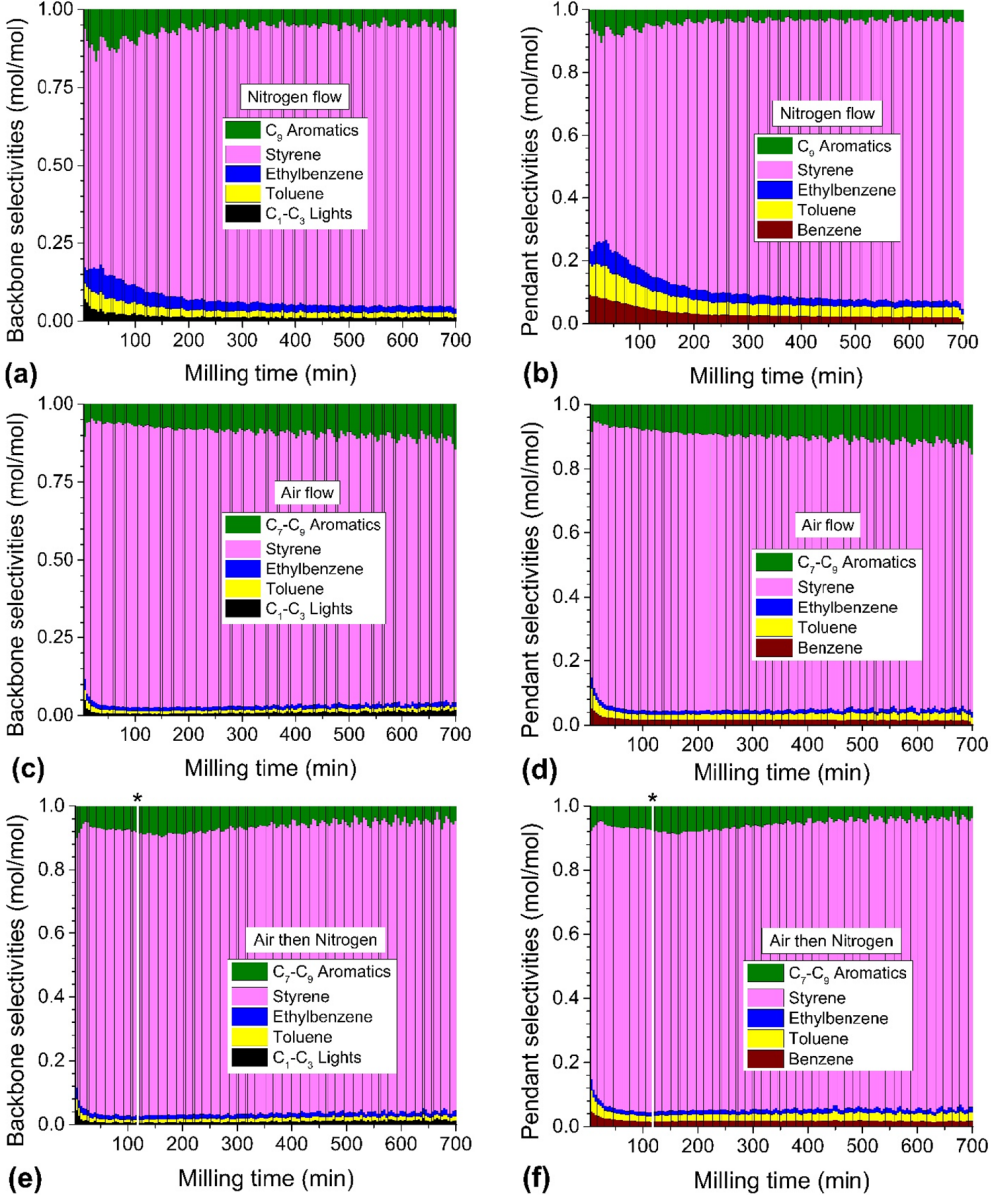
Depolymerization of 1 g of PS inside a 25 mL stainless steel reactor with eight 10 mm stainless steel balls under a continuous purge gas flow rate of 60 mL/min. Stacked bar graphs for selectivities (a, c, e) calculated with respect to backbone carbons and (b, d, f) calculated with respect to pendant phenyl groups for N_2_, air, and air then N_2_ flow, respectively. White line under asterisk in (e, f) denotes instance when air was switched to N_2_.

The time-evolution of selectivities was notably different under air (Figure 3c,d). Unlike the gradual evolution toward an apparent steady-state selectivity profile under N_2_, there was a trend toward increasing selectivity for C_7_–C_9_ aromatics. The C_7_ oxygenate benzaldehyde made up a majority of this selectivity fraction (see magnitudes of instantaneous yields in Figure S6b,g,h in SI). For the experiment where air was switched to N_2_ at 120 min, the trend of increasing selectivity for oxygenates followed the expected profile under air up to the point of the switch. The elevated C_7_–C_9_ selectivity persisted until 160 min before reversing and subsequently trending toward the steady-state selectivities observed under N_2_. This means that the response was delayed by 40 min in the selectivity profile from the elimination of O_2_ from the system. The production of oxygenates like benzaldehyde also persisted for up to 6 h beyond the switch to N_2_ (Figure S6b, SI), which indicates that oxygen reacts with the residual polymer to an extent that lasts beyond its presence in the bulk gas phase.

Under air, the elevated selectivities for C_1_–C_3_ gases, benzene, toluene and ethylbenzene in the initial hour of milling decreased more rapidly with time compared to milling in N_2_. With respect to absolute amounts of these products, instantaneous yields under N_2_ and air were approximately the same for the first 80–120 min (see Figure S6a,e,f in SI). Afterward, a sharper decline in flow occurred under air for all the minor products. This suggests that the yields of light gases and some aromatics, such as toluene and ethylbenzene, are correlated and affected equally by the presence of O_2_. Benzene was an exception (Figure S6d, SI), which saw increased flow under air for the first 120 min before experiencing a decline such that by 160 min a higher yield was sustained under N_2_ than air.

Evidence of the chemical role of O_2_ was obtained with ESR spectroscopy. The evolution of radical concentrations with milling time was monitored by sampling residues milled for 60, 120, 240, and 480 min. Carbon- and oxygen-centered organic radicals appeared as one sharp signal at a g-value of ca. 2.002 which is equivalent to ca. 3360 G in magnetic field (see Figures S8 and S9 in SI). The intensity of the signal is a qualitative measure of the radical concentration when normalized by sample mass. The relative radical concentrations with milling time confirmed that a higher concentration of stable radicals was sustained under air compared to under N_2_ at all times (Figures 4b and S11 in SI). Under air, the free radical population increased sharply until 240 min but appeared to plateau and by 480 min had decreased slightly. Under N_2_, the population of radicals increased more steadily until 480 min at which point the population was still less than 60% of the maximum recorded radical concentration (at 240 min under air). Comparing the measured radical populations to the styrene flow data, it was observed that in air or N_2_, the point of maximum monomer production (120 min under air and 210 min under N_2_) occurred at only around 30% of the peak radical population. In the styrene flows (Figure 2c) leading up to the maximum in monomer production, there was a linear increase in styrene, with the ultimate maximum styrene flow under air being a little more than twice the maximum value under N_2_ (see overlay of these data in Figure S7b).

**Figure 4:**
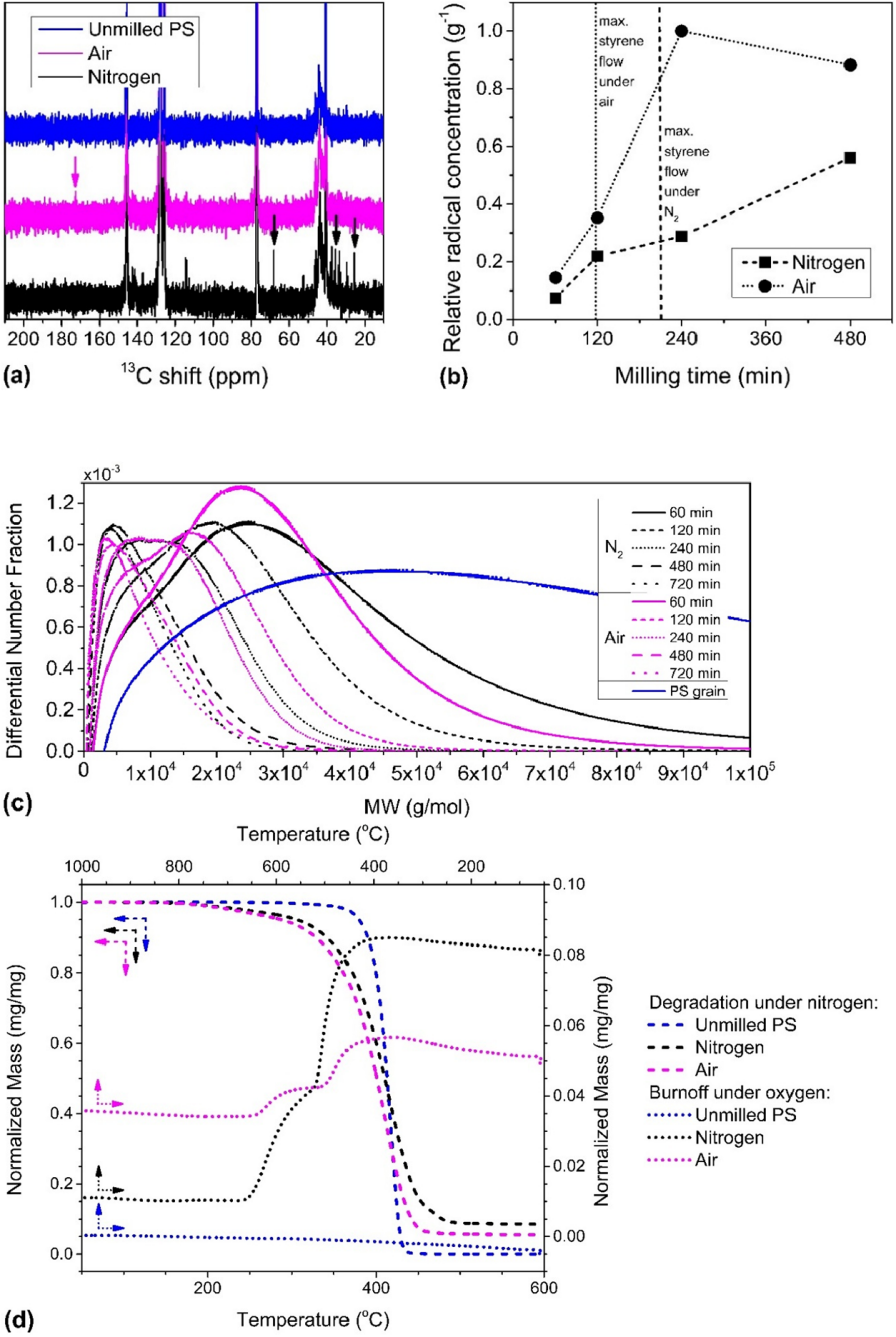
(a) ^13^C NMR spectra of PS residue milled for 720 min inside a 25 mL stainless steel reactor with eight 10 mm stainless steel balls under a continuous purge gas (N_2_ or air) flow rate of 60 mL/min; new moieties compared to unmilled PS are indicated by downward arrows. (b) concentration of free radicals calculated from ESR spectra intensities in residues milled under air and N_2_ normalized to the highest amount measured across all samples. (c) differential number fraction molecular weight distributions in linear scale for PS residues recovered after milling under N_2_ or air atmosphere obtained from SEC calculated from PS standards. (d) TGA degradation curves for the same residues first under N_2_ atmosphere to thermally degrade the polymer followed by burnoff under O_2_ atmosphere to combust leftover mass.

The MW distributions of PS residues milled for 60, 120, 240, 480, and 720 min are depicted in Figure 4c under N_2_ and air (also Figure S1b,c). In both cases, significant shifts to lower MWs by several orders of magnitude predominantly occurred while *M_n_* was higher than 10,000 g/mol, which corresponds to a degree of polymerization of around 100.^22^ It is well-known that below this characteristic *M_n_* of 10,000 g/mol, the chemical process of MW reduction through chain scission becomes less favorable energetically compared to the physical rearrangement of the polymer because the intermolecular van der Waals interactions become weaker than C–C bond energies in the backbone.^23^ Under both air and N_2_, a prominent shoulder toward low MW developed within the first 120 min of grinding. The shape of the curves (at 60, 120 min) indicated preferential fragmentation of longer chains in the distribution.^24,25^ The shoulders had disappeared by 480 min with half of the distribution lying below the characteristic *M_n_* of 10,000 g/mol.

To assess chemical changes in PS residues, ^13^C NMR spectra were recorded on samples recovered after 720 min of milling under N_2_ and air atmospheres (Figure 4a). Compared to unmilled PS, the residue from milling under N_2_ does exhibit a number of new peaks primarily in the aliphatic region. Only the aliphatic peak near 30 ppm had been discussed previously for samples milled up to 6 h.^12^ New peaks at 114 ppm and around 126 ppm (in between the characteristic chemical shifts of the phenyl carbons of PS) could be indicative of residual styrene monomer (see Figure S2b, SI). In the NMR spectrum of PS milled under air, a small peak at 173 ppm can be assigned to the ester group, implying oxidation of PS chains, possibly via a Baeyer–Villiger mechanism as was reported in mechanochemical PE cracking.^16^ This reaction path would indicate a high concentration of peroxy or oxy radicals on the PS particles, which is corroborated by the saturation of the radical population under air observed in ESR. PS milled under air exhibited practically no new features in the aliphatic region, and only weak signals at the 114 (olefin) and 126 (aromatic) ppm were observed, which point to persistent styrene absorbed in the residue.

Minor products such as toluene, ethylbenzene, allylbenzene and C_1_–C_3_ hydrocarbon gases whose hydrogen-to-carbon ratio is greater than that of styrene (H/C = 1:1) were observed persistently among volatile products, especially under N_2_. The removal of more hydrogen than carbon from PS during milling, therefore, leaves behind more dehydrogenated carbonaceous products like coke. These could also be responsible for the NMR signals at 114 and 126 ppm which are present in both N_2_ and air-milled residue.

Degradation curves of the residues in TGA, depicted in Figure 4d, show earlier onset of degradation in both residues compared to unmilled PS. In the sample milled under N_2_, this can be attributed to increased prevalence of weak aliphatic links, as corroborated by the aliphatic signals in the NMR spectrum of the residue. For the air-milled sample, lower degradation temperatures were observed due to reduced thermal stability of oxidized linear PS compared to the regular polymer.^26^ Unmilled PS degraded completely under N_2_ and left no residual mass. PS milled under N_2_ on the other hand formed a residue of as much as 8% of its initial mass, which can be combusted under O_2_ in a subsequent heating cycle, consistent with the behavior of coke.^27,28^ PS milled under O_2_ also had 5% residual mass during degradation under N_2_, and subsequent combustion removed an additional 1% of the initial mass. The residual masses after degradation cannot be immediately attributed to coke, because during the long duration of milling, steel shavings from mechanical attrition of the reactor and grinding spheres could accumulate in the residue. To exclude the mass contribution of steel shavings, TGA of degradation under N_2_ was also conducted on residue redried from the solution NMR samples (Figure S5, SI), since preparation of NMR samples included filtering out insoluble shavings. The degradation curves obtained were similar to those in Figure 4b, and similar residual masses (4%) from both N_2_ and air-milled samples were obtained. This further supports the formation of soluble coke species during ball milling of PS, which manifest as a distinctive yellow tint in organic solutions of the residues (Figure S15).

## Discussion

By combining observations of depolymerization product flow rates with properties of the PS residue sampled discretely at various durations of milling, three kinetic regimes or stages can be identified, where the dominant phenomena that drive depolymerization reactions are distinct (Figure 5). However, because mechanochemical driving forces operate at the material rather than molecular scale,^29^ the same mechanisms are expected to be operative across all stages. We shall discuss the stages chronologically and introduce the mechanisms during the exposition of the first stage.

**Figure 5:**
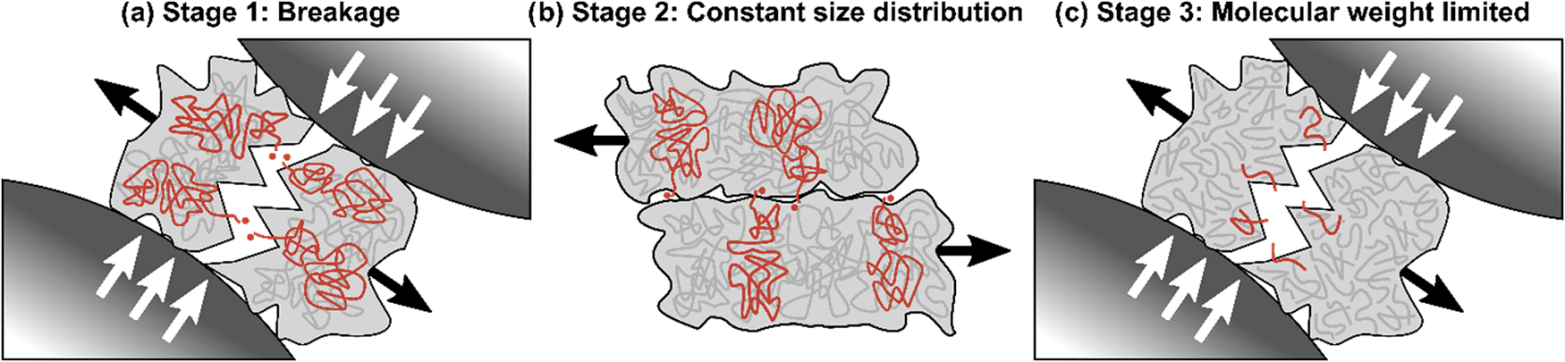
Illustrations of the dominant macroscopic mechanism responsible for depolymerization in each of the three stages of kinetics.

### Stage 1: Breakage-Coupled Depolymerization

Particle size analysis based on microscopy images (Figure S13) indicates that within 120 min of milling, particles initially greater than 100 μm in size were reduced to 2–40 μm (Figure S13c,d). Since particle breakage results in strain on individual polymer chains, a significant number of bonds are broken in the backbone, and mechanoradicals are formed. This leads to a drastic reduction of MW (Figure 5a) and the formation of chain end radicals.^12^ During this stage of grinding, styrene flow reached its peak under air (Figures 2c and S6c), and the yields of minor hydrocarbon products (C_1_–C_3_ gases, aromatics) all trended upward and attained peak flow under both air and N_2_ (Figure S6i in SI). The persistent radical population also grew steadily (Figure 4b). Therefore, the first stage of depolymerization is characterized by the creation of macroscopic surfaces populated by mechanoradicals due to irreversible breakage of PS particles, which lasts from the start of milling to around 120 min, when PS particles cease to break further (Figure S14a).

The mechanochemical genesis of most of reactive intermediates in PS is the chain scission step converting a backbone bond (A) to a pair of chain end radicals, one secondary (B) and one primary (C) radical, as depicted starting from the boxed structure in Figure 6. The subsequent depolymerization step (from species B) that yields styrene is thermodynamically limited because its reverse reaction is favored under standard conditions. However, the depolymerization equilibrium can be shifted by purging styrene from the reactor.^21^ In this context, it is important to analyze the temperature-sensitivity of the depolymerization reaction. While the reactor temperature rises from the ambient and equilibrates to ca. 50 °C upon the start of milling,^12^ it is equilibrated in as little as 40 min.^12^ In contrast, all the product flows grew continuously for 120 min. Thus, a variable other than temperature is a significant driver of the observed kinetics.

**Figure 6:**
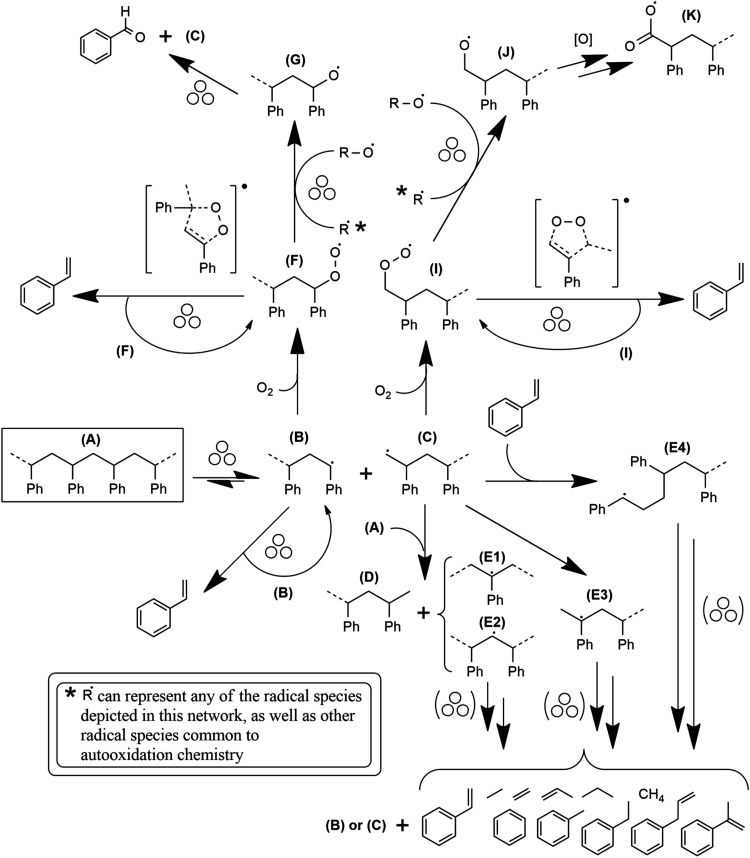
Proposed reaction network in mechanochemical PS depolymerization under inert and oxidizing atmospheres. The progenitor species (A) for the whole network is boxed. Arrows accompanied by three circles indicate steps suggested to be driven mechanochemically whereas arrows without the symbol indicate spontaneous reactions that may occur outside of grinding collisions. Since the focus is on steps leading to the formation of volatile products, radical termination steps such as disproportionation are omitted for simplicity.

During this stage when particle breakage is coupled to product flow, new surfaces are created constantly by breaking large particles into smaller ones, so the frequency of the scission reaction A → B + C (Figure 6) is expected to scale with fresh surface area created on any given particle from a grinding action.^30,31^ Along with this initiation step, complicated networks of spontaneous radical reactions following scission should also increase in frequency.^32^ The density of radicals generated through surface creation is sufficiently high^33–35^ to allow for the liberation of volatile small molecules by a single macroscopic mechanical action, such as slicing.^36,37^ It is no surprise then that a variety of volatile hydrocarbon products should form unselectively (see Figure 3a,b) from the complex array of radical intermediates present under these transient conditions. Thus, the creation of fresh surfaces through particle breakage is the predominant phenomenon driving the formation of small hydrocarbon molecules other than styrene.

For some of the hydrocarbon byproducts, an explicit reaction pathway to their formation can be constructed from mechanisms present at thermal pyrolysis conditions.^38–40^ At first, one might attribute the formation of benzene and C_1_–C_3_ hydrocarbon gases (regardless of presence of O_2_) to the dealkylation of substituted aromatic products like toluene, styrene, ethylbenzene and allylbenzene. However, even if this reaction path is relevant, dealkylation of small molecules cannot be the sole production source of benzene and light gases because the ratio of carbon atoms of C_1_–C_3_ gases to benzene molecules in the products is expected to be 2 for dealkylation, whereas the actual value was less than 1 at all instants (see Figures S6a versus S6d, SI). Instead, the presence of benzene points to elimination reactions involving the phenyl pendant groups on the PS chain^41^ that occur in tandem with decomposition of the remaining backbone via a variety of radical transfer reactions, as is observed during thermal pyrolysis of PS above 360 °C.^42^ In pyrolysis mechanisms, the formation of hydrocarbons such as methane, toluene, and ethylbenzene requires chain fragmentation from a radical site on the interior of the PS chain. Such radical sites can only be formed from intramolecular or intermolecular transfer reactions of a terminal radical generated via chain scission. For example, methane results from an intramolecular hydrogen abstraction step by primary scission radicals.^43^ Such radical transfer reactions lead to the gradual appearance of new aliphatic carbon moieties on the milled PS not present initially, which is confirmed in the NMR spectra of PS residue milled under N_2_. Representative radical intermediates E1–4 are depicted in the bottom half of Figure 6. Under N_2_, B is mainly responsible for monomer production whereas the less stable C is more prone to undergoing competing reactions, such as hydrogen abstraction to form more stable midchain secondary radical species such as E1–2, or hydrogen shift to form E3.^43^

The presence of O_2_ should have no effect on the breakage rate of PS particles, so the different kinetic behavior observed under air must be due to reactions of oxygen species with the fresh PS surfaces as they formed to suppress the production of minor hydrocarbon products. This is expected to occur through autoxidation^44^ of chain end radicals, depicted in the conversion of the secondary radical B to peroxy radical species F and of the primary radical C to species I in Figure 6. These reactions are highly favored even at cryogenic conditions,^30^ so O_2_ can be seen as a reagent that prolongs the lifetime of radical species originating from chain scission reactions – a radical trapping agent. The same milling duration at the same set of operating conditions should generate the same number of radical pairs from chain scission regardless of the atmosphere. Therefore, the higher concentration of radicals observed under air suggests that O_2_ stabilizes the secondary radical B and the primary radical C, so that species F and I become sites of monomer production. Such mechanisms are consistent with those present in oxidative pyrolysis reactions.^45^

Since new aliphatic carbon moieties were hardly present in measurable concentrations in the residue milled under air (Figure 4a), and these aliphatic carbon moieties arise from radical transfer reactions, it is natural to conclude that O_2_ also mitigated the migration of radicals from the chain end to the interior of chains, thereby simplifying the depolymerization mechanisms to favor higher levels of initial styrene production at chain ends.^45^ As depicted in the top half of Figure 6, we suggest the peroxy radical chain ends F and I yield styrene in a concerted step instigated by grinding (a mechanochemically driven reaction), for example, one involving a five-membered ring transition state. The similarity of the styrene flow curves under sustained air flow and air flow for only the first 120 min indicates that an appreciable population of species F and I is established early in the process and that these intermediates continue to contribute to styrene monomer formation for an extended time.

In addition to particle breakage, two other surface chemical phenomena are operative during ball mill grinding: viscoelastic sintering of particles which destroys surfaces^46^ and frictional interaction between particle surfaces.^47^ The former is not significant in the current kinetic regime, in which breakage dominates. However, frictional interactions, which do not involve creation or destruction of surfaces, are expected to dissipate kinetic energy into a form that can be used to drive chemical reactions. Because friction between particles brings their surfaces into transient mechanical contact, different surface radical species located on any two distinct particles can be brought into sufficiently close physical proximity for a chemical reaction to occur. For example, frictional contacts that bring together a carbon radical and a peroxy radical lead to coupling followed by disproportionation,^48^ which is an event we expect to occur with significant frequency with increasing radical population while milling in air. This friction-driven disproportionation of peroxy radicals F and I will transform them into oxy radicals G and J (as depicted in Figure 6). The formation of benzaldehyde, the most prominent oxygenated aromatic, is attributed to a mechanochemical β-elimination reaction from species G. Alternatively, one could hypothesize a similar cyclic transition state involving a highly reactive trioxyl radical intermediate from species G.^48–50^ Either way, mechanistic paths leading to the formation of benzaldehyde also funnels polymeric radicals toward the formation of the primary oxy radical species J, which can neither react via β-elimination nor via a cyclic transition state. The expected oxy elimination product from species J is formaldehyde, which was not observed in any experiment. Thus, species J is mechanochemically less active than its secondary counterpart G. Instead of undergoing mechanochemically driven reactions, species J can be further oxidized in a series of nonmechanochemical reactions to the carboxyl radical K, whose resonance stabilization allows it to accumulate on the PS residue. The accumulation of species K accounts for the larger radical population present under air detected by ESR (Figure 4b) which does not sustain higher styrene production, as well as the eventual observation of the ester group in the ^13^C NMR spectrum of the same residue (Figure 4a).

### Stage 2: Depolymerization at Constant Size Distribution

By the end of the first stage at around 120 min, the average particle size inside the ball mill had reached its equilibrium value (Figure S14a), and smaller, more numerous particles cause collision forces to be distributed over a wider area of mechanical contact compared to concentrated stress points when particle sizes are still large.^51,52^ At this point, nearly all byproduct flows slowed down (Figure S6a–f in the SI), but under N_2_, styrene flow continued to rise until attaining and then sustaining at a near-constant maximum value starting around 210 min (Figure 2c), and the steady state selectivity profile was established around the same time (Figure 3a,b). The radical concentration measured by ESR continued to increase in this stage, while the MW distribution shifted toward shorter chains more gradually compared to the earlier regime. Thus, the start of the second stage is characterized by attainment of an observed limit in average particle size that cannot be decreased further by more grinding, coinciding with reduction in the flow of aromatic hydrocarbon byproducts but continued increase in flow of styrene under N_2_.

Since the particle size distribution stayed approximately constant while impacting forces from the ball mill remained unchanged,^12^ it is clear that particle breakages creating fresh surfaces are counterbalanced by viscoelastic sintering of particles that destroy existing surfaces.^46^ The absence of net particle size reduction does not mean that radical creation and propagation are discontinued, because breakage still occurs albeit in dynamic equilibrium with sintering, and this breakage can still activate polymer chains via scission – along with the subsequent reactions that give rise to hydrocarbon byproducts, hence the continuation of the mechanisms illustrated in the bottom half of Figure 6. However, if breakage is the only means by which small molecules are produced, we should expect the styrene flow to remain correlated with the (decreasing) flow of the rest of the byproducts as is the case during the first stage. Instead, styrene flow continued to increase beyond 120 min under N_2_ (Figure S14b) while the flows of all byproducts (C_1_–C_3_ gases and aromatic hydrocarbons) reached their peak flow at this time (Figure S14c). Thus, the reduced selectivities of byproducts (Figure 3a,b) in favor of styrene indicate a transition from breakage to a different mechanochemical driving force that favors the production of styrene.

In discussing the previous kinetic regime, we had identified breakage, sintering and friction as three distinct mechanical phenomena of chemical consequence occurring in the ball mill to varying levels of significance. From attainment of the equilibrium particle size around 120 min of milling (Figure S14a), we have identified sintering as a more significant phenomenon in this stage of kinetics relative to the earlier one. Likewise, the change in the flow trends of byproducts occurring around the same time (Figure S14c) shows an increased significance of frictional interactions between the surfaces of polymer particles compressed in between metal grinding surfaces during collisions (Figure 5b) while PS powder is milled at the equilibrium particle size distribution. Since the surface area scales as the square of particle size, it is logical that reduced particle sizes relative to the breakage regime result in more frictional interactions and more monomer production via this phenomenon.

To illustrate how the same mechanisms depicted in Figure 6 can lead to the production of small molecules under frictional surface interactions, we shall first consider the product flows under N_2_. As was discussed in the previous kinetic regime, the rapid and irreversible creation of fresh macroscopic surfaces during particle breakage leads to a high local density of free radical species on these surfaces. In an inert atmosphere, these radicals cannot attain further stability by reaction with O_2_ in the gas phase, rather, they are expected to undergo tandem reactions due to the physical proximity of individual radical species. If a sufficient energy density is imparted on a local domain, chain scission and the production of volatile small molecules can occur during a single impact, with the consequence being that all volatile product flows are correlated and trend in the same direction. This was indeed observed to be the case (see Figure S14b,c in SI).

In going from the breakage into the frictional stage, there is a growing pool of persistent but still reactive secondary radical species (Figure 4b) such as B and E1–4. Since the relative stability of these secondary radicals affords them a longer lifetime that may exceed the mean free time of a PS particle between consecutive mechanochemical collisions, a radical generated during one impact may persist until the next impact on the same particle to yield monomers. These radicals can be viewed as the source of steady styrene production in the frictional stage (especially following peak flow at around 210 min), and among them species B is suggested to be the most important since it yields styrene directly via β-elimination. Unlike what would be expected in the breakage stage, the observed gradual decline in the flow of byproducts such as benzene, toluene and ethylbenzene throughout the frictional stage did not cause any similar decline in styrene flow, because most of the reaction networks responsible for the formation of hydrocarbon byproducts also recover species B, from which styrene production may occur so long as the radical species can persist to encounter multiple collisions. In summary, while breakage continues to occur in a dynamical equilibrium with sintering in this stage, the production of styrene through breakage is less significant than in the first stage as indicated by the trend of styrene flow no longer following the trend of byproduct flows in this second kinetic regime.

In the presence of O_2_, the second regime is characterized by the same mechanical behavior in PS particles, but styrene flow declined gradually (following the trend of declining byproduct flows) rather than attaining a plateau beyond 120 min. The primary reason is that unlike with N_2_, the reaction network involving O_2_ does not lead back to species B. During the breakage stage, the presence of O_2_ allows species B and C formed on freshly created PS surfaces to be rapidly converted to peroxy radicals F and I, which are persistent yet still sufficiently reactive to yield styrene directly while regenerating themselves. By the frictional stage, a significant density of these species is expected on all the PS particle surfaces generated in the previous stage. As particle sintering and breakage equilibrate, the PS surfaces closed by sintering are those covered by peroxyl species rather than a clean hydrocarbon surface as would be the case under inert N_2_. Subsequent particle breakage is therefore more likely to proceed along the previously closed surfaces, due to obvious chemical differences.

While the same oxidized surfaces are encapsulated and re-exposed upon sintering and breakage, disproportionation of peroxy radicals F and I during both friction and rebreakage will transform them into oxy radicals G and J.^48^ Reactions involving species G all lead to the primary oxy radical J, which has been deemed mechanochemically inactive for small molecule production and instead lead to the relatively inert carboxyl radical K. The growth in abundance of species K in turn causes the steady decline of more reactive radical species over time, resulting in gradually diminishing styrene flow. The accumulation of relatively inactive radical species K–and others like it–does not require the continued presence of O_2_ in the bulk gas phase, but only the continued redistribution of oxygen radicals already incorporated into the chemical structure of the PS chains via primary oxy radical species like J. This explains why exposure of PS to O_2_ only in the first 2 h of milling causes sustained flows of hydrocarbon monomers that all follow the same trajectory as continuous exposure to O_2_, even after benzaldehyde production had ceased. Some of the oxygen atoms are incorporated into the polymer irreversibly. Therefore, with progressive accumulation of species K, the oxidized PS residue also becomes more mechanochemically inert, so the greater yield of styrene seen in the first stage grinding in an oxidizing atmosphere is an inherently transient phenomenon.

### Stage 3: Reaching the Limiting Molecular Weight

In spite of the continuation of depolymerization at constant particle size distribution beyond 480 min, transition from the second stage to a distinct third stage of kinetics is proposed to occur when MW degradation has progressed to the extent that a significant fraction of chains is shorter than the characteristic *M_n_* of 10,000 g/mol (Figure 5c). Mechanically, the additional flexibility of an individual chain in PS below the limiting MW of 10,000 g/mol allows the chain to move uniformly in response to the applied force from a collision, thus decreasing the probability for any part of the chain to experience a significant localized, intrachain strain for homolytic C–C cleavage.^23^

With respect to the mechanisms depicted in Figure 6, it can be understood that the reduction of the MW distribution to below 10,000 g/mol reduces the frequency of mechanochemical chain cleavage events. This occurs starting at around 480 min, when monomer flow under both N_2_ and air commence a gradual decline at about the same rate (Figure 2d). Since this behavior is observed under both inert and oxidative atmospheres, it can be considered a distinct kinetic regime that occurs at extended milling times (beyond 480 min), when mechanochemical C–C cleavage becomes a diminishing source of new radical species regardless of whether these are carbon- or oxygen-centered radicals, all because an ever-growing fraction of chains in the population are below the threshold MW for efficient chain scission. However, since conversion of a chain segment to monomers requires at least two elementary steps, and only the first step of scission is limited by the characteristic MW, chains that are too short to undergo further scission may still undergo depolymerization at thermodynamically appropriate conditions^21^ provided that a radical remains from a previous stage or is formed by alternative means, such as hydrogen abstraction by another molecule. In fact, due to the decreasing trend of the (positive) depolymerization enthalpy with decreasing chain length in olefinic polymers,^53,54^ depolymerization from a shorter chain is less energy demanding from a low MW feedstock.

## Conclusions

In this work, ball milling of PS with purge gas flow coupled to in-line analysis of the effluent using GC-FID was leveraged to gain detailed kinetic insight and mechanistic understanding of PS depolymerization using mechanochemistry. Three major kinetic regimes in the depolymerization reaction of this commodity polyolefin are revealed, in which the reaction is controlled primarily by (in chronological order) particle breakage, friction, and the limiting molecular weight of the residue. The transition between the breakage and friction kinetic regimes is identified based on trends of byproduct flows coinciding with the evolution of particle sizes with increased milling time (Figure S14c), while the molecular weight-limited regime is observed by comparing styrene flows under oxidizing and inert atmospheres (Figure 2d).

In an inert atmosphere, depolymerization reactions are initially dependent on particle breakages and the creation of fresh PS surfaces, which results in the formation of mechanoradicals followed by styrene production alongside aromatic hydrocarbon (mainly benzene, toluene, ethylbenzene, allylbenzene) and C_1_–C_3_ (mainly methane, ethene, propene) byproducts. Observation of byproducts whose H:C ratio is greater than that of styrene implies limited coke formation which is corroborated by TGA of residues. Styrene flow becomes steady and the selectivity toward styrene is higher (over 90%, which is better than thermal and catalytic pyrolysis approaches^55^) when friction overtakes surface creation as the dominant driver of depolymerization, before the overall rate of monomer production declines at extended milling times when the limiting MW for mechanoradical formation from PS is reached at 10,000 g/mol. The increased time resolution of the product analysis allows for delineating these kinetic regimes, whereas the rate of styrene production had appeared to be constant in prior work on PS depolymerization.^12^ Practically speaking, this operating regime (characterized by depolymerization with limited changes in particle size) is suitable for developing and testing mechanochemical catalysts for depolymerization reactions, since it allows for delineating the chemistry at the catalyst–feedstock interface from physicochemical transformations of the feedstock.

The presence of O_2_ is the determining factor for styrene production throughout the first two regimes. Oxygen radical species facilitate the formation of monomers from chain ends, whereas under N_2_ the radicals tend to migrate further along the PS chain before terminating. This causes the formation of many new carbon environments in the PS residue as was shown by NMR. The role of O_2_ in suppressing radical migration should be mechanistically translatable to other olefinic polymers due to common free radical mechanisms. While the cumulative yield of byproducts under O_2_ is lower, a wider variety of byproducts is formed including oxygenated monoaromatics (mainly benzaldehyde) in addition to hydrocarbons. ESR spectroscopy of the residues shows that a higher concentration of radicals is maintained in the presence of O_2_ compared to pure N_2_, consistent with the conversion of carbon-centered scission radicals to more persistent peroxy, oxy and eventually carboxyl radicals.

Overall, our study illustrates kinetic limitations to mechanochemical PS recycling at various stages of the process. An oxidative atmosphere is on the whole more conducive to attaining the highest possible yield of styrene in the shortest milling time, with the highest styrene flows obtained during the particle breakage regime. Thus, mechanochemical reactor designs that allow for high breakage rates and the smallest possible equilibrium particle sizes are suitable for harnessing the beneficial kinetics of this transient regime. The low intrinsic mechanochemical reactivity of PS (and even lower for other polyolefins) may be improved by utilizing reactor designs which enable a higher density of frictional interactions, adding small amounts of radical starters, and most importantly by engineering reaction microenvironments with a higher local temperature to overcome the thermodynamic limits of the depolymerization step.^21^ Finally, the inherent limiting MW for polyolefin chain scission will inevitably reduce mechanoradical formation by direct chain scission, but even so, monomer production may continue from preexisting radicals or radicals introduced onto short chains via mechanisms such as hydrogen abstraction, provided that localized high-temperature transient environments are generated in mechanochemical collisions. The continuous addition of unprocessed feedstock may be able to manage the rate at which mechanoradicals are formed and utilized by short chains.

## Supplementary Material



## Notes

Normalized full-range MWDs of PS samples determined by size exclusion chromatography in logarithmic scale and tabulated average MWs; NMR spectra originals of styrene, unmilled PS and residues; auxiliary TGA curves of residues, instantaneous product yield versus time curves for PS ball milled under different atmospheres; ESR analysis procedure and sample spectra; microscopy and miscellaneous images (PDF)

## References

[1] Vollmer I., Jenks M. J. F., Roelands M. C. P., White R. J., van Harmelen T., de Wild P., van der Laan G. P., Meirer F., Keurentjes J. T. F., Weckhuysen B. M. (2020). Beyond Mechanical Recycling: Giving
New Life to Plastic Waste. Angew. Chem., Int.
Ed..

[2] Aydonat S., Hergesell A. H., Seitzinger C. L., Lennarz R., Chang G., Sievers C., Meisner J., Vollmer I., Göstl R. (2024). Leveraging
Mechanochemistry for Sustainable Polymer Degradation. Polym. J..

[3] Rizzo A., Peterson G. I. (2024). Progress toward Sustainable Polymer
Technologies with
Ball-Mill Grinding. Prog. Polym. Sci..

[4] Štrukil V. (2021). Highly Efficient
Solid-State Hydrolysis of Waste Polyethylene Terephthalate by Mechanochemical
Milling and Vapor-Assisted Aging. ChemSusChem.

[5] Tricker A. W., Osibo A. A., Chang Y., Kang J. X., Ganesan A., Anglou E., Boukouvala F., Nair S., Jones C. W., Sievers C. (2022). Stages and Kinetics of Mechanochemical Depolymerization
of Poly­(Ethylene Terephthalate) with Sodium Hydroxide. ACS Sustainable Chem. Eng..

[6] Lee H. W., Yoo K., Borchardt L., Kim J. G. (2024). Chemical Recycling of Polycarbonate
and Polyester without Solvent and Catalyst: Mechanochemical Methanolysis. Green Chem..

[7] Tricker A. W., Hebisch K. L., Buchmann M., Liu Y. H., Rose M., Stavitski E., Medford A. J., Hatzell M. C., Sievers C. (2020). Mechanocatalytic
Ammonia Synthesis over TiN in Transient Microenvironments. ACS Energy Lett..

[8] Fantozzi N., Volle J.-N., Porcheddu A., Virieux D., García F., Colacino E. (2023). Green Metrics in Mechanochemistry. Chem. Soc. Rev..

[9] Jubinville D., Esmizadeh E., Saikrishnan S., Tzoganakis C., Mekonnen T. (2020). A Comprehensive Review
of Global Production and Recycling
Methods of Polyolefin (PO) Based Products and Their Post-Recycling
Applications. Sustainable Mater. Technol..

[10] Martín A. J., Mondelli C., Jaydev S. D., Pérez-Ramírez J. (2021). Catalytic
Processing of Plastic Waste on the Rise. Chem.

[11] Balema V. P., Hlova I. Z., Carnahan S. L., Seyedi M., Dolotko O., Rossini A. J., Luzinov I. (2021). Depolymerization of Polystyrene under
Ambient Conditions. New J. Chem..

[12] Chang Y., Blanton S. J., Andraos R., Nguyen V. S., Liotta C. L., Schork F. J., Sievers C. (2024). Kinetic Phenomena
in Mechanochemical
Depolymerization of Poly­(Styrene). ACS Sustainable
Chem. Eng..

[13] Jung E., Yim D., Kim H., Peterson G. I., Choi T. (2023). Depolymerization of
Poly­(A-methyl Styrene) with Ball-mill Grinding. J. Polym. Sci..

[14] Hergesell A. H., Seitzinger C. L., Burg J., Baarslag R. J., Vollmer I. (2025). Influence
of Ball Milling Parameters on the Mechano-Chemical Conversion of Polyolefins. RSC Mechanochem..

[15] Jung E., Cho M., Peterson G. I., Choi T. (2024). Depolymerization of Polymethacrylates
with Ball-Mill Grinding. Macromolecules.

[16] Nguyen V. S., Chang Y., Phillips E. V., DeWitt J. A., Sievers C. (2023). Mechanocatalytic
Oxidative Cracking of Poly­(Ethylene) Via a Heterogeneous Fenton Process. ACS Sustainable Chem. Eng..

[17] Li L., Vozniuk O., Cao Z., Losch P., Felderhoff M., Schüth F. (2023). Hydrogenation
of Different Carbon Substrates into Light
Hydrocarbons by Ball Milling. Nat. Commun..

[18] Li L., Leutzsch M., Hesse P., Wang C., Wang B., Schüth F. (2025). Polyethylene
Recycling via Water Activation by Ball
Milling. Angew. Chem., Int. Ed..

[19] Hergesell A. H., Popp S., Meena R., Ospina Guarin V. M., Seitzinger C., Sievers C., Li G., Vollmer I. (2025). Homolytic
Fracture of Inorganic Crystalline Materials Enhances the Mechano-Chemical
Degradation of Polypropylene. Chem. Sci..

[20] Hergesell A. H., Baarslag R. J., Seitzinger C. L., Meena R., Schara P., Tomović Ž., Li G., Weckhuysen B. M., Vollmer I. (2024). Surface-Activated Mechano-Catalysis for Ambient Conversion
of Plastic Waste. J. Am. Chem. Soc..

[21] Chang Y., Nguyen V. S., Hergesell A. H., Seitzinger C. L., Meisner J., Vollmer I., Schork F. J., Sievers C. (2024). Thermodynamic
Limits of the Depolymerization of Poly­(Olefin)­s Using Mechanochemistry. RSC Mechanochem..

[22] Staudinger H., Heuer W. (1934). Über Hochpolymere
Verbindungen, 93. Mitteil.: Über
Das Zerreißen Der Faden-Moleküle Des Poly-styrols. Ber. Dtsch. Chem. Ges. A B Ser..

[23] Sohma J. (1989). Mechanochemistry
of Polymers. Prog. Polym. Sci..

[24] Nguyen T. Q. (1994). Kinetics
of Mechanochemical Degradation by Gel Permeation Chromatography. Polym. Degrad. Stab..

[25] Jimenez-Francisco M., Carrillo J. G., Garcia-Cerda L. A. (2021). Mechanochemical
Tuning of Molecular
Weight Distribution of Styrene Homopolymers as Postpolymerization
Modification in Solvent-Free Solid-State. J.
Appl. Polym. Sci..

[26] Cameron G. G., McWalter I. T. (1982). Thermal Degradation of Polystyrene—4. Decomposition
of Oxygen-Containing Polymers. Eur. Polym. J..

[27] Pradhan A. R., Wu J. F., Jong S. J., Tsai T. C., Liu S. B. (1997). An Ex Situ
Methodology for Characterization of Coke by TGA and 13C CP-MAS NMR
Spectroscopy. Appl. Catal., A.

[28] Minh, C. Le. ; Li, C. ; Brown, T. C. Kinetics of Coke Combustion during Temperature-Programmed Oxidation of Deactivated Cracking Catalysts. In Studies in Surface Science and Catalysis; Elsevier, 1997; Vol. 1997, pp 383–390.

[29] Michalchuk A. A. L., Boldyreva E. V., Belenguer A. M., Emmerling F., Boldyrev V. V. (2021). Tribochemistry, Mechanical Alloying, Mechanochemistry:
What Is in a Name?. Front. Chem..

[30] Backman D. K., Devries K. L. (1969). Formation of Free
Radicals during Machining and Fracture
of Polymers. J. Polym. Sci., Part A-1.

[31] Butyagin P. Y., Berlin A. A., Kalmanson A. E., Blyumenfeld L. A. (1960). Macroradicals
in the Mechanical Degradation of Polymers in the Glassy State. Rubber Chem. Technol..

[32] Kruse T. M., Woo O. S., Wong H. W., Khan S. S., Broadbelt L. J. (2002). Mechanistic
Modeling of Polymer Degradation: A Comprehensive Study of Polystyrene. Macromolecules.

[33] Zhurkov S. N., Zakrevskyi V. A., Korsukov V. E., Kuksenko V. S. (1972). Mechanism of Submicrocrack
Generation in Stressed Polymers. J. Polym. Sci.,
Part A-2.

[34] Zhurkov S. N., Korsukov V. E. (1974). Atomic Mechanism of Fracture of Solid
Polymers. J. Polym. Sci.: Polym. Phys. Ed..

[35] Zhurkov S. N., Kuksenko V. S. (1975). The Micromechanics of Polymer Fracture. Int. J. Fract..

[36] Regel’ V. R., Muinov T. M. (1966). Mass-Spectrometry
Method of Studying the Kinetics of
the Evolution of Volatile Products from Polymers under Load. Polym. Sci. U.S.S.R..

[37] Amelin A. V., Muinov T. M., Pozdnyakov O. F., Regel V. R. (1970). Comparison of the
Mass Spectra of the Volatile Products Liberated from Polymers in the
Course of Mechanical Destruction and Thermal Degradation. Polym. Mech..

[38] Nisar J., Ali G., Shah A., Iqbal M., Khan R. A., Sirajuddin, Anwar F., Ullah R., Akhter M. S. (2019). Fuel Production from Waste Polystyrene via Pyrolysis:
Kinetics and Products Distribution. Waste Manage..

[39] Huang J., Li X., Meng H., Tong H., Cai X., Liu J. (2020). Studies on
Pyrolysis Mechanisms of Syndiotactic Polystyrene Using DFT Method. Chem. Phys. Lett..

[40] Karunarathna B., Wanniarachchi J. D., Prashantha M. A. B., Govender K. K. (2023). Enhancing Styrene
Monomer Recovery from Polystyrene Pyrolysis: Insights from Density
Functional Theory. J. Mol. Model..

[41] Bouster C., Vermande P., Veron J. (1989). Evolution
of the Product Yield with
Temperature and Molecular Weight in the Pyrolysis of Polystyrene. J. Anal. Appl. Pyrolysis.

[42] Carniti P., Beltrame P. L., Armada M., Gervasini A., Audisio G. (1991). Polystyrene Thermodegradation. 2.
Kinetics of Formation
of Volatile Products. Ind. Eng. Chem. Res..

[43] Guyot A. (1986). Recent Developments
in the Thermal Degradation of Polystyrene-A Review. Polym. Degrad. Stab..

[44] Ingold K. U. (1969). Peroxy
Radicals. Acc. Chem. Res..

[45] Chien, J. C. W. ; Kiang, J. K. Y. Pyrolysis and Oxidative Pyrolysis of Polypropylene. In Advances in Chemistry; Allara, D. L. ; Hawkins, W. L. , Eds.; ACS Publications, 1978; pp 175–197.

[46] Lin Y. Y., Hui C. Y., Jagota A. (2001). The Role of Viscoelastic Adhesive
Contact in the Sintering of Polymeric Particles. J. Colloid Interface Sci..

[47] Colacino E., Carta M., Pia G., Porcheddu A., Ricci P. C., Delogu F. (2018). Processing and Investigation
Methods
in Mechanochemical Kinetics. ACS Omega.

[48] Lee R., Coote M. L. (2016). Mechanistic
Insights into Ozone-Initiated Oxidative
Degradation of Saturated Hydrocarbons and Polymers. Phys. Chem. Chem. Phys..

[49] Semes’ko D. G., Khursan S. L. (2008). Quantum-Chemical
Calculations of the Structure of Trioxyl
Radicals. Russ. J. Phys. Chem. A.

[50] Cerkovnik J., Eržen E., Koller J., Plesničar B. (2002). Evidence for
HOOO Radicals in the Formation of Alkyl Hydrotrioxides (ROOOH) and
Hydrogen Trioxide (HOOOH) in the Ozonation of C–H Bonds in
Hydrocarbons 1. J. Am. Chem. Soc..

[51] Molina-Boisseau S., Le Bolay N. (2000). Size Reduction
of Polystyrene in a Shaker Bead Mill-Kinetic
Aspects. Chem. Eng. J..

[52] Molina-Boisseau S., Le Bolay N. (2002). Characterisation of
the Physicochemical Properties
of Polymers Ground in a Vibrated Bead Mill. Powder Technol..

[53] Jessup R. S. (1948). The Heat
and Free Energy of Polymerization of Ethylene. J. Chem. Phys..

[54] Dainton F. S., Ivin K. J. (1950). Changes of Entropy and Heat Content during Polymerization. Trans. Faraday Soc..

[55] Kumar V., Khan A., Rabnawaz M. (2022). Efficient Depolymerization of Polystyrene
with Table Salt and Oxidized Copper. ACS Sustainable
Chem. Eng..

