# Supplementary material for: Stages of Mechanochemical Depolymerization of Poly(styrene) Powder in Oxidative and Inert Atmospheres
Source: ACS Sustain Chem Eng. 2025 Oct 26;13(44):18970–82. doi: 10.1021/acssuschemeng.5c05942 (PMC12606713; doi:10.1021/acssuschemeng.5c05942)
Supplement: Supplementary file 1 [file sc5c05942_si_001.pdf]

Supplementary Information

Stages of Mechanochemical Depolymerization of Poly(styrene) Powder in Oxidative and Inert Atmospheres

Yuchen Chang,[1+] Adrian H. Hergesell,[2+] Claire L. Seitzinger,[2] Aubrey M. Hepstall,[1]
Ina Vollmer[2] and Carsten Sievers[1]*
[1] School of Chemical & Biomolecular Engineering, Georgia Institute of Technology, Atlanta, Georgia, 30332 (U.S.A.)
[2] Inorganic Chemistry and Catalysis, Institute for Sustainable and Circular Chemistry, Utrecht University, Universiteitsweg 99, 3584 CG, Utrecht, The Netherlands
**carsten.sievers@chbe.gatech.edu*
[+]authors contributed equally to experimental results

Number of pages: 20
Number of figures: 15
Number of tables: 2

S.A. Size Exclusion Chromatography (SEC)

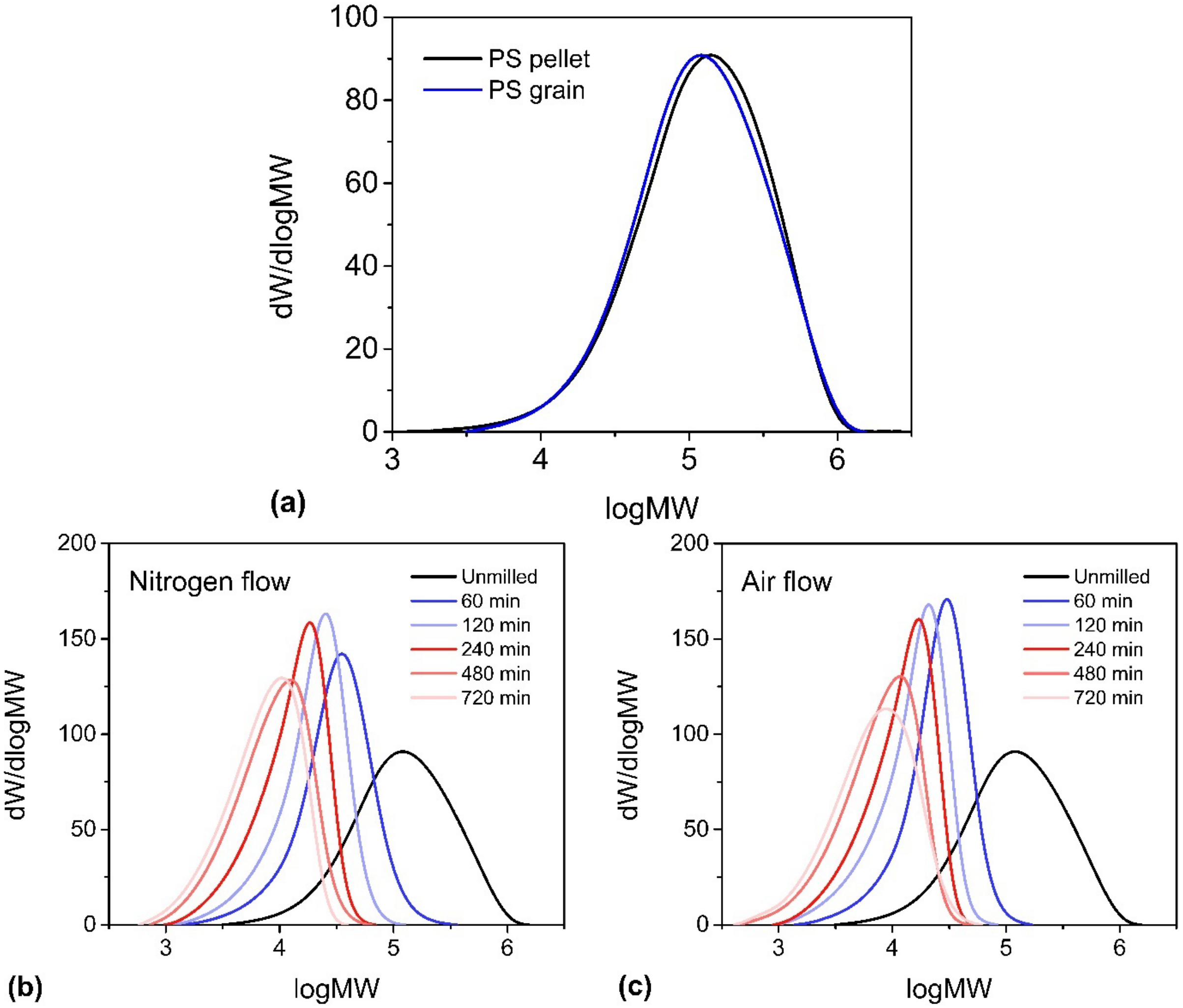

Figure S1. Log-scale molecular weight distributions of (a) unmodified PS pellet as purchased and crushed PS grains of the same material used for depolymerization experiments – indicating an insignificant broadening of the distribution peak in the crushed material, and of PS residues recovered after milling for various amounts of time in flowing N_2 (b) and air (c) atmospheres.

Table S1. Number (M_N) and weight (M_W) average molecular weights and dispersion calculated from molecular weight distributions in Figure S1b-c.

Time (min)	0 (unmilled)	60	120	240	480	720
Nitrogen flow						
M_n	85,873	22,284	14,378	9,617	5,482	4,718
M_w	213,867	39,553	23,296	15,309	9,675	9,041
Highest MW	1,544,150	358,476	126,401	69,223	63,304	39,577
Lowest MW	3,154	1,335	1,087	972	722	579
Dispersity	2.490	1.775	1.620	1.592	1.765	1.916
Air flow						
M_n	85,873	19,230	11,117	8,828	6,340	5,261
M_w	213,867	29,787	17,937	13,910	10,787	8,847
Highest MW	1,544,150	167,919	83,606	55,380	50,239	60,737
Lowest MW	3,154	1,388	942	894	436	407
Dispersity	2.490	1.549	1.613	1.576	1.701	1.682

S.B. Nuclear Magnetic Resonance (NMR) Spectroscopy

^{13}C spectra were collected in the range -20 ppm < δ_C < 220 ppm with 4096 scans per spectra and calibrated to the $CDCl_3$ triplet centered at δ_C = 77.23 ppm.

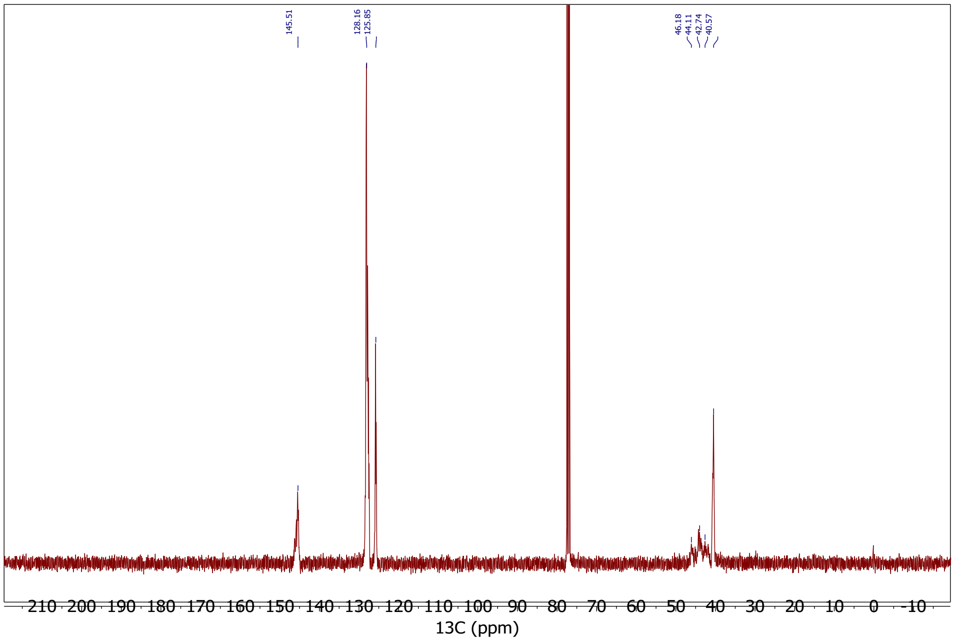

Figure S2a. Unmilled PS; ^{13}C NMR (101 MHz, $CDCl_3$)
δ 40.57, 42.74, 44.11, 46.18, 125.85, 128.16, 145.51 (PS)

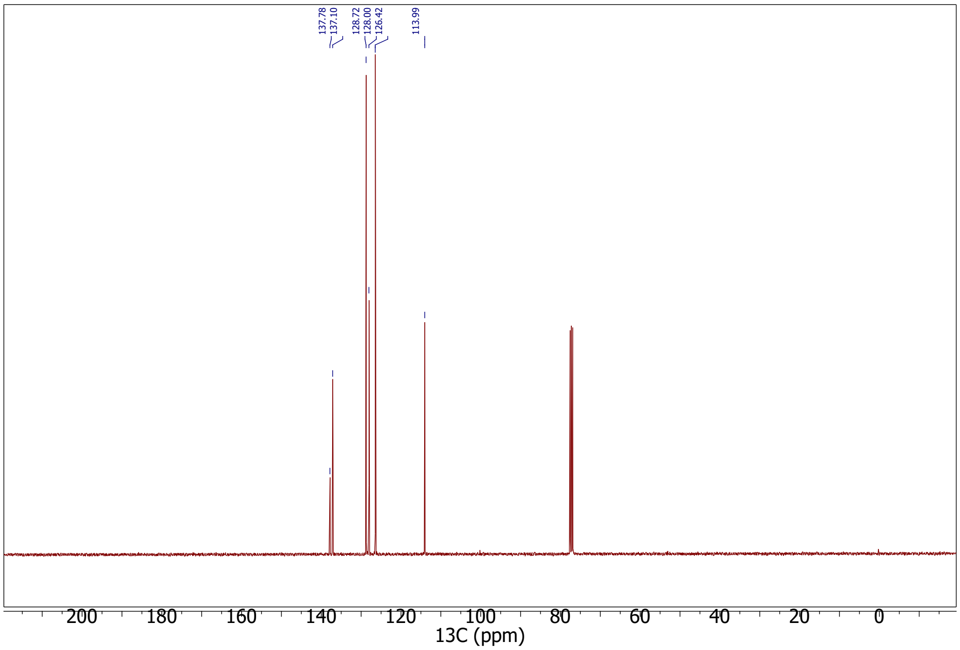

Figure S2b. Styrene standard; ^{13}C NMR (101 MHz, $CDCl_3$)
δ 113.99, 126.41, 127.99, 128.71, 137.09, 137.78 (styrene)

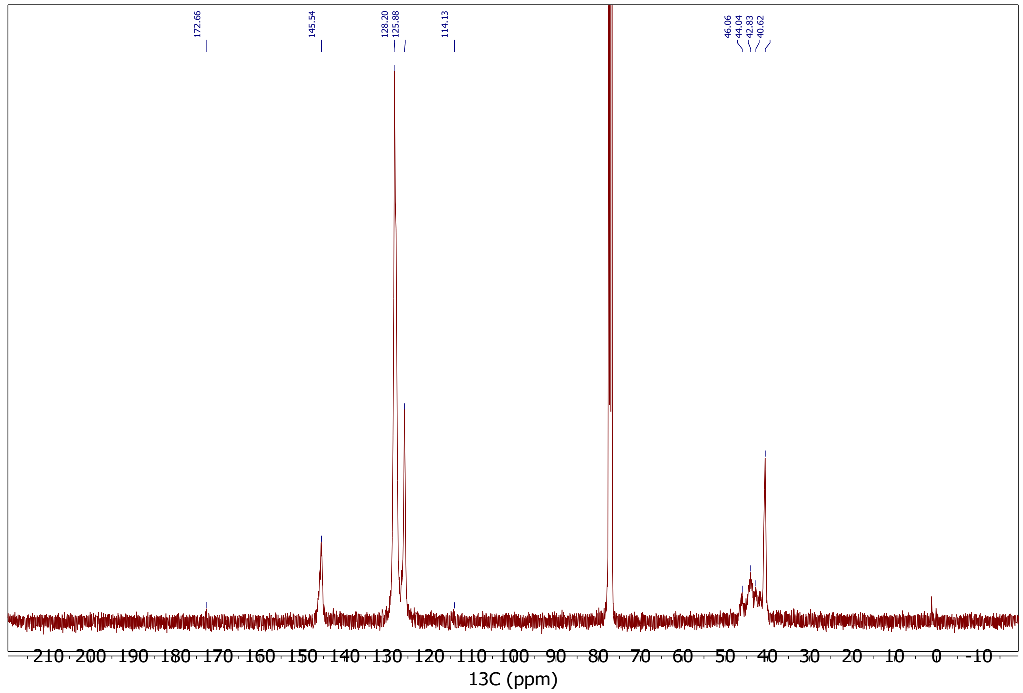

Figure S3. PS milled for 720 minutes under air atmosphere; ^{13}C NMR (101 MHz, $CDCl_3$) δ 40.62, 42.83, 44.04, 46.06, 125.88, 128.20, 145.54 (PS); 114.13 (styrene); 172.66 (carboxyl)

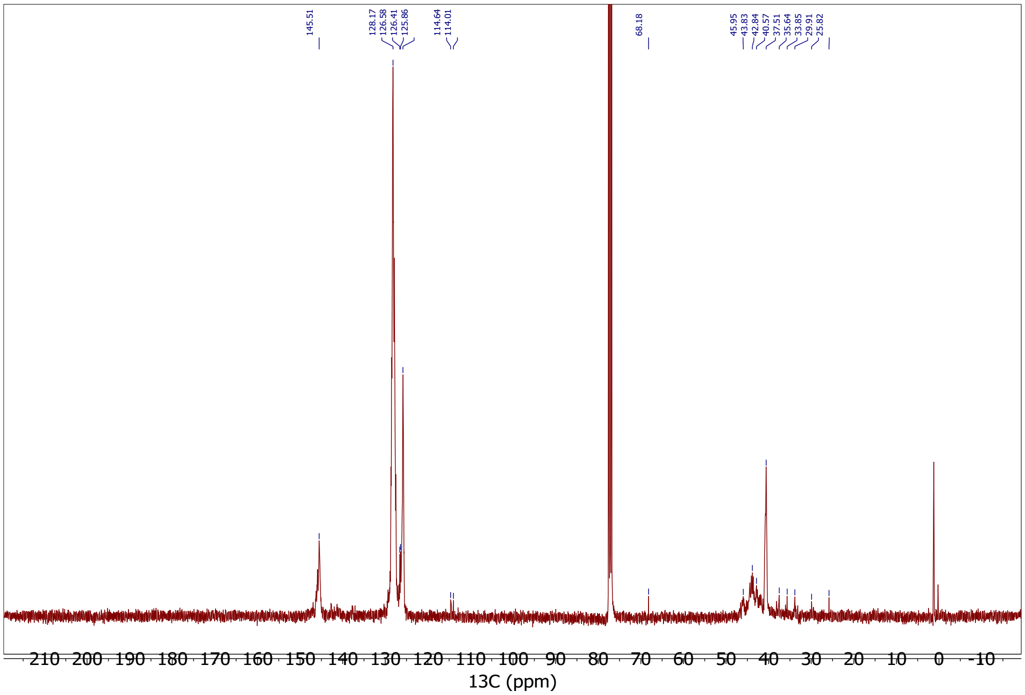

Figure S4. PS milled for 720 minutes under nitrogen atmosphere; ^{13}C NMR (101 MHz, $CDCl_3$) δ 40.57, 42.84, 43.83, 45.95, 125.86, 128.17, 145.51 (PS); 25.82, 29.91, 33.85, 35.64, 37.51 (aliphatic); 68.18 (alkyne or amine); 114.01, 126.41 (styrene); 114.64 (olefin); 126.58 (aromatic)

S.C. Thermogravimetric Analysis (TGA)

Auxiliary TGA experiments were conducted on dried solid residue recovered from samples used in NMR experiments (Figure S5). TGA was performed on a Thermal Advantage Instruments SDT Q600 in alumina crucibles. Degradation curves were obtained using about 10 mg of residue at a flow rate of 20 mL/min of N_2, with a temperature program starting at ambient conditions (20°C) and ramping up to 600°C at a heating rate of 10°C/min.

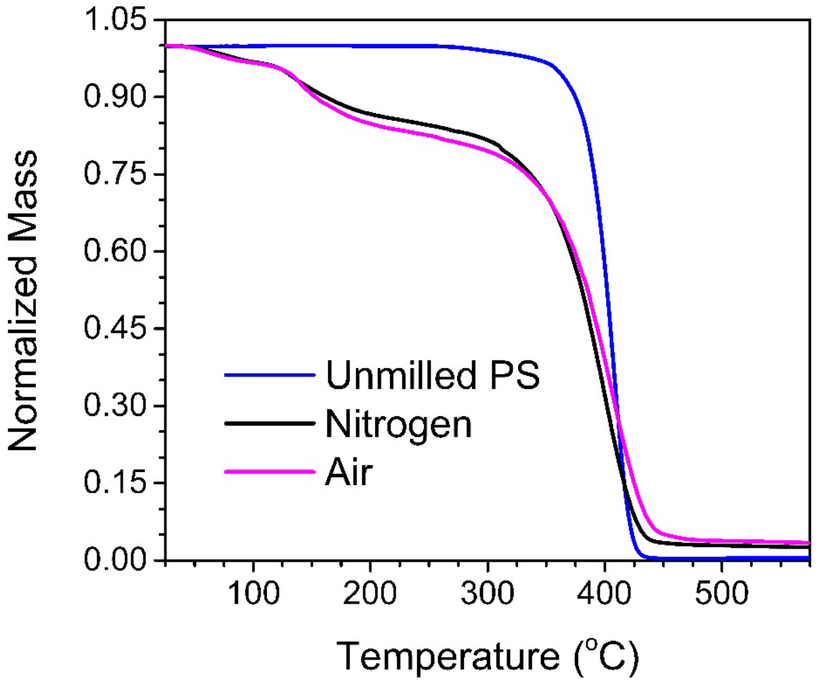

Figure S5. TGA curves for unmilled PS and residues recovered from NMR experiments milled under N_2 and air for 720 minutes. Mass loss in degradation curves of the residues before 200°C were due to residual organic solvent (tetrahydrofuran and deuterated chloroform) in the samples; actual mass of residues could be estimated at 85% of the total.

S.D. Yields and Selectivities with Time

Instantaneous yields of volatile depolymerization products in effluent gas stream calculated from in-line GC downstream from reactor.

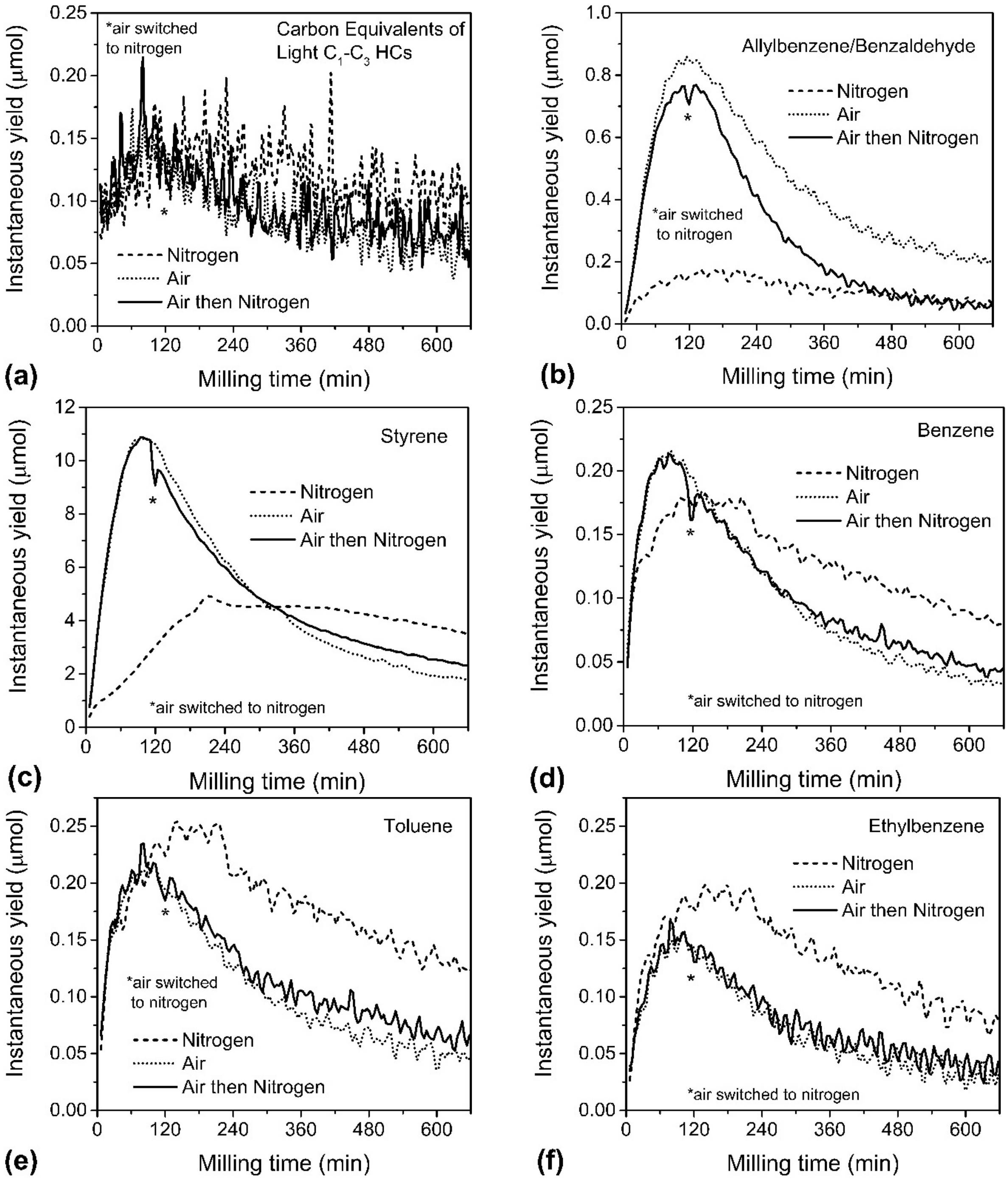

Figure S6. Instantaneous molar yields (a)-(f) of individual and lumped products from PS powder ball milled under N2 and air atmospheres.

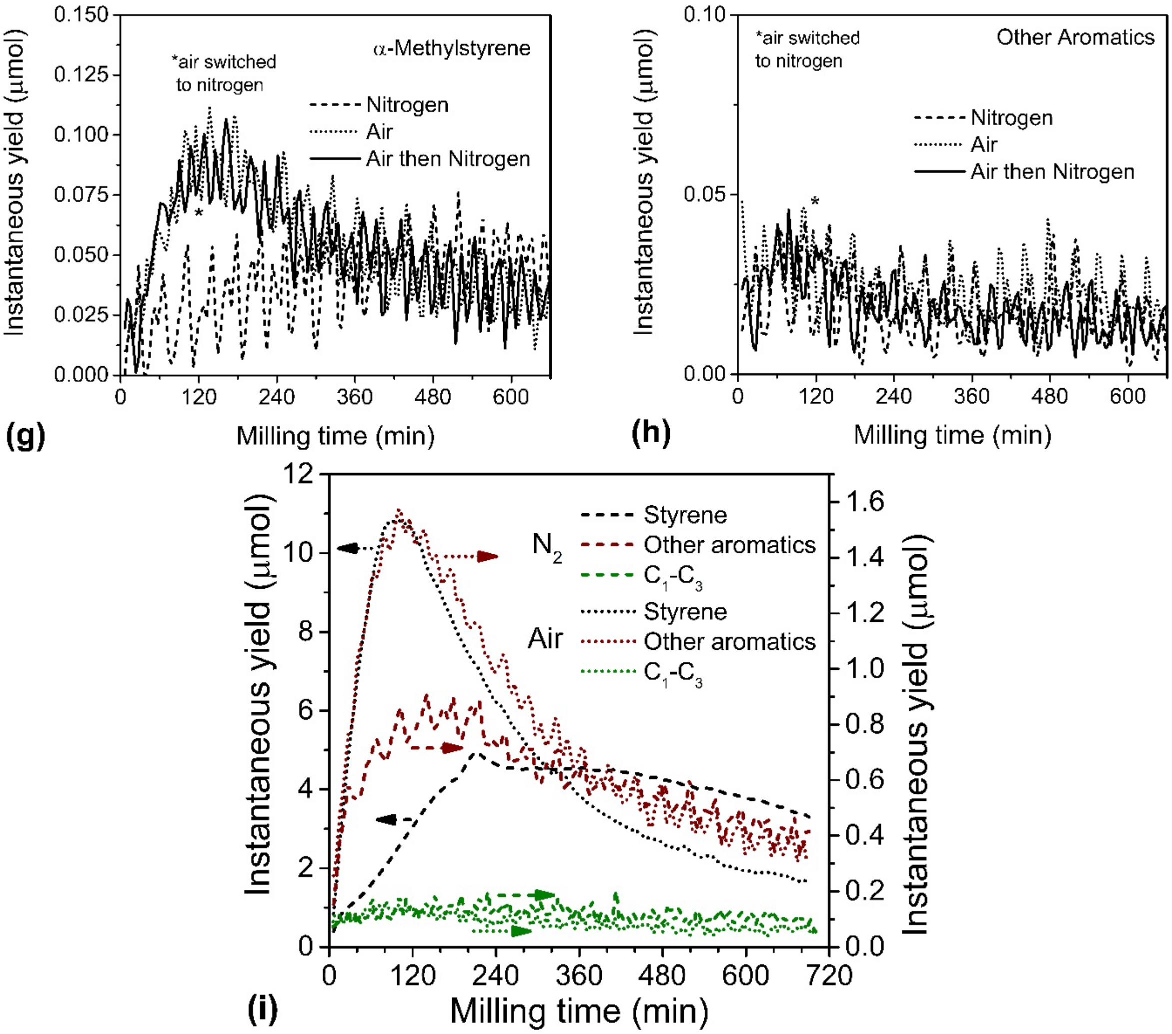

Figure S6 cont'd. Instantaneous molar yields (g)-(h) of individual and lumped products, (i) of styrene, the sum of other aromatics and the sum of all C_1-C_3 hydrocarbon gases from PS powder ball milled under N_2 and air atmospheres.

On the GC system used in this study, the elution times of allylbenzene and benzaldehyde are nearly the same which made resolution of the peaks difficult; for simplicity, the yield is plotted for the two products combined in Figure S6b. The presence of a second product other than allylbenzene under air is apparent in that yields of no other aromatic hydrocarbon byproduct differed so greatly in magnitude (four times at peak) between air and nitrogen atmospheres. It is apparent that by switching from air to nitrogen at 120 min, production of benzaldehyde persists up to 440 min before matching the (baseline) yield of allylbenzene under N_2.

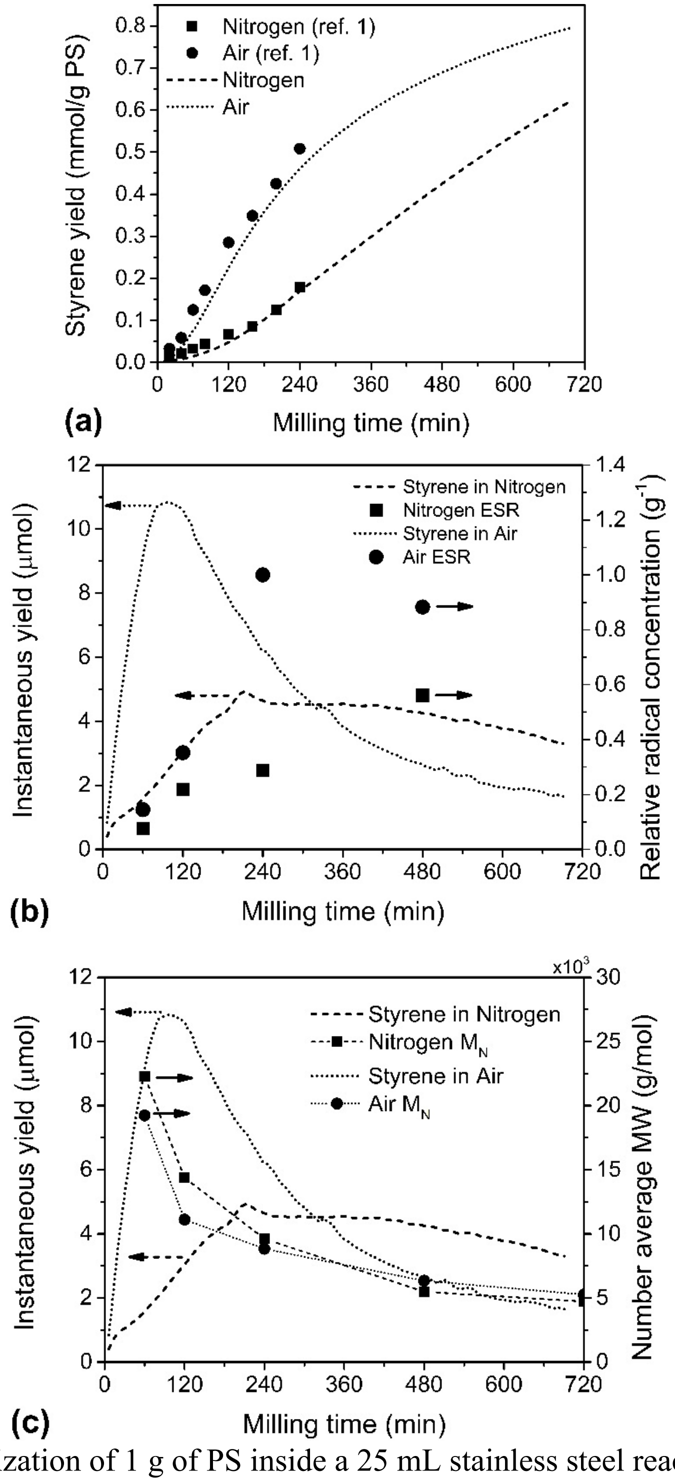

Figure S7. Depolymerization of 1 g of PS inside a 25 mL stainless steel reactor with eight 10 mm stainless steel balls in N_2 or air atmospheres; (a) styrene yields from Figure 2a in main text plotted alongside styrene yields reported in Ref. 1, indicating excellent agreement with data collected on a similar setup; (b) instantaneous yields from Figure S6c plotted with ESR measurements from Figure 4b and (c) the same instantaneous yields with M_N values in Table S1.

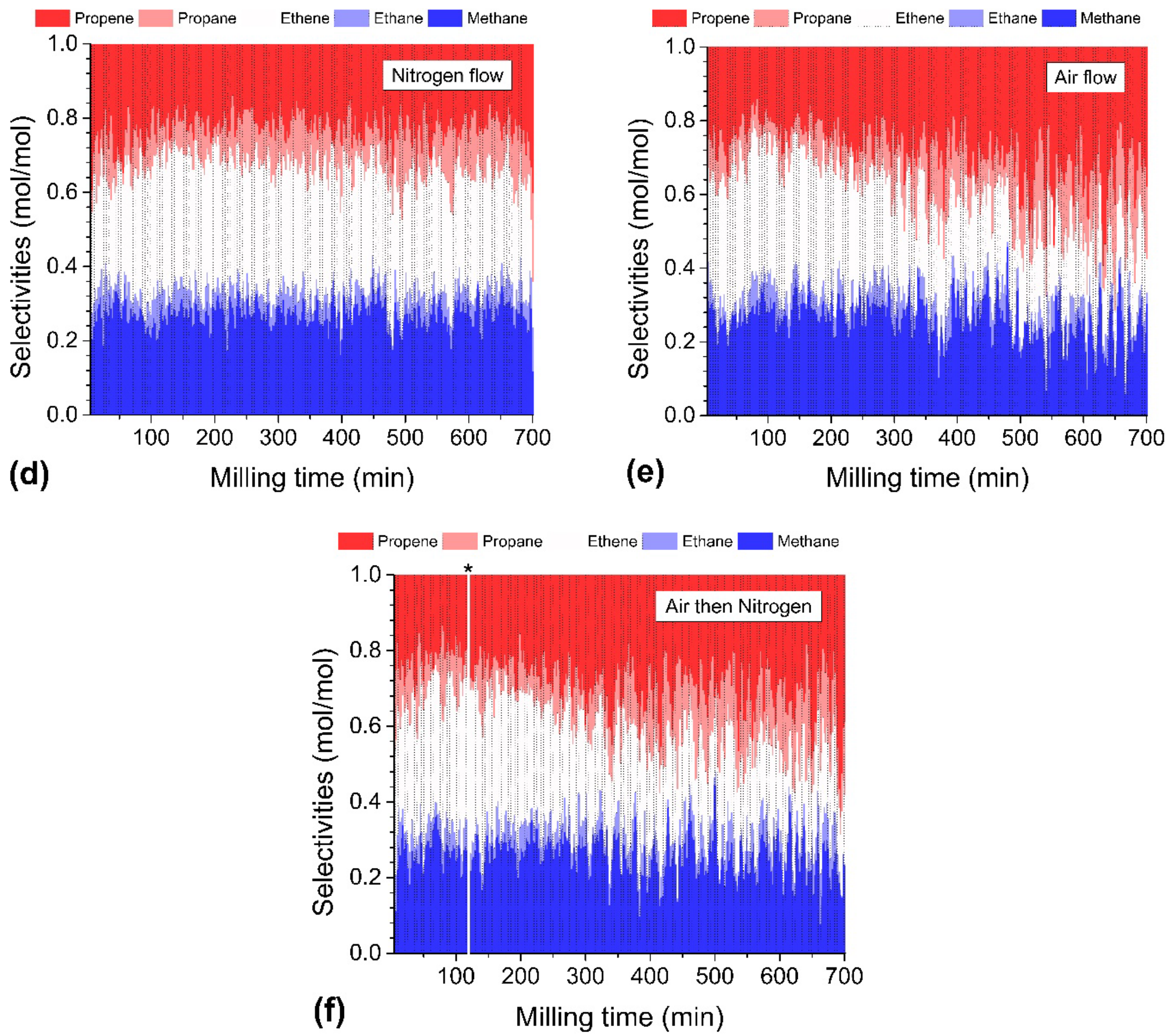

Figure S7 cont'd. Stacked bar graphs for instantaneous selectivities of C_1-C_3 hydrocarbon gases in 1 g of PS milled inside a 25 mL stainless steel reactor with eight 10 mm stainless steel balls under a continuous flow rate of 60 mL/min of (d) N_2, (e) air and (f) air switched to nitrogen. White line under asterisk in (f) denotes instance when air was switched to N_2. These selectivities were calculated with respect to the instantaneous yields plotted in Figure S6a, excluding aromatic products.

Table S2. Cumulative yields of individual products in PS milled under N_2 or air at select instances in time. Yields are expressed in units of mg/g PS (initial mass).

Time (min)	60	120	240	480	720
Nitrogen flow					
Methane	0.006	0.013	0.031	0.062	0.085
Ethane	0.002	0.006	0.014	0.024	0.033
Ethene	0.011	0.028	0.070	0.136	0.183
Propane	0.005	0.012	0.028	0.061	0.092
Propene	0.015	0.032	0.068	0.130	0.184
Benzene	0.13	0.32	0.71	1.27	1.62
Toluene	0.19	0.47	1.11	2.07	2.71
Ethylbenzene	0.17	0.43	1.00	1.81	2.29
Styrene	1.58	5.00	17.78	44.31	64.97
Cumene	0.02	0.05	0.09	0.17	0.24
Allylbenzene	0.13	0.36	0.91	1.67	2.13
n-Propylbenzene	0.01	0.03	0.05	0.09	0.12
α-Methylstyrene	0.03	0.07	0.18	0.49	0.79
Air flow					
Methane	0.006	0.015	0.029	0.050	0.062
Ethane	0.002	0.006	0.010	0.018	0.023
Ethene	0.014	0.035	0.066	0.100	0.120
Propane	0.005	0.012	0.021	0.045	0.068
Propene	0.014	0.031	0.062	0.113	0.158
Benzene	0.17	0.40	0.74	1.09	1.24
Toluene	0.20	0.47	0.89	1.35	1.61
Ethylbenzene	0.14	0.35	0.70	1.05	1.24
Styrene	8.00	23.40	48.04	71.71	82.95
Cumene	0.03	0.07	0.12	0.21	0.31
Allylbenzene & Benzaldehyde	0.59	1.91	4.40	7.04	8.46
n-Propylbenzene	0.02	0.04	0.07	0.13	0.17
α-Methylstyrene	0.06	0.18	0.47	0.83	1.04

S.E. Electron Spin Resonance (ESR) Spectroscopy

ESR spectra of milled residue contain two main contributions:

1. A sharp signal around $g = 2.002$ at a magnetic field of ca. 3360 G represents a mixture of carbon-centered radicals generated via mechanochemical scission of polystyrene chains and oxygen-centered radicals produced by oxidation of those carbon-centered radicals.
2. A broad signal spanning from 0 to 6000 G is observed in some cases. The most extreme case is depicted in Figure S8. In addition, a smaller broad signal with a peak close to 3200 G is observed in some cases (see Figure S9), which also overlaps with the sharp signal of organic radicals at $g = 2.002$. These two background signals are attributed to oxidized iron impurities in the samples generated by abrasion of the milling equipment due to forceful collisions. For example, nanometer-sized oxidized iron structures in the enzyme ferritin feature very broad ESR signals spanning over several thousands of G.[2]

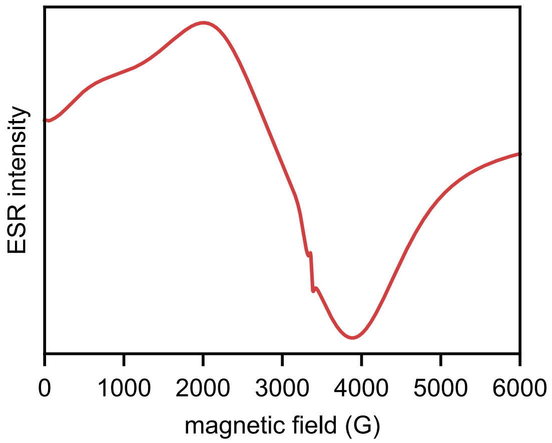

Figure S8. Full-range mass-normalized ESR spectrum after milling 1 g PS for 240 min under N_2 atmosphere.

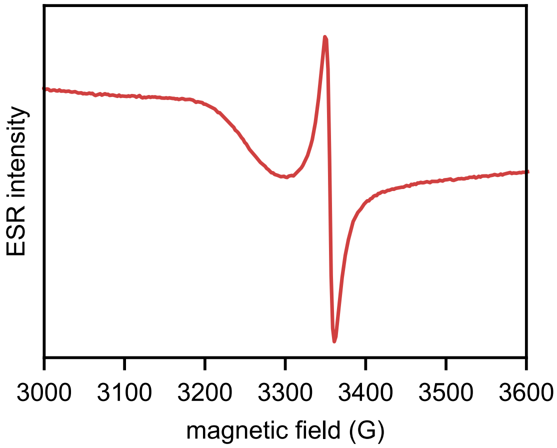

Figure S9. Mass-normalized ESR spectrum after milling 1 g PS for 120 min under N_2 atmosphere.

We performed iron-free reference experiments with ZrO_2 grinding spheres in a WC jar and confirmed a virtually flat baseline with a single organic signal at $g = 2.002$ (see Figure S10). Therefore, we focus our further analysis on the isolation and quantification of this signal.

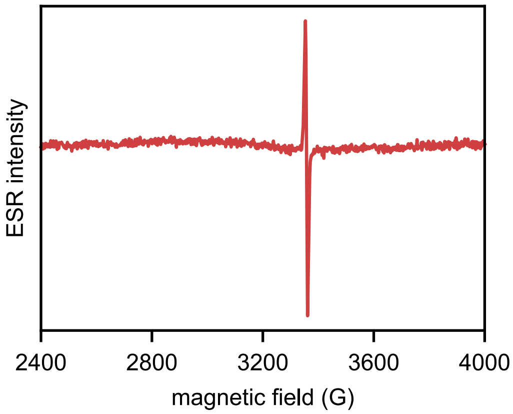

Figure S10. Mass-normalized ESR spectrum after milling 1 g PS for 60 min under N_2 atmosphere.

We performed four different analyses to assess the relative radical amounts in the sample. Prior to analysis, all spectra were normalized by sample mass and receiver gain. Subsequently, the following five approaches were used to calculate the values plotted in Figure 4b in the main text, all leading to similar results in terms of radical amounts vs. time. Data processed using the 5 methods individually are plotted in Figure S11.

1. To obtain the signal amplitude as a measure for radical amounts, we subtracted the minimum from the maximum intensity in a magnetic field window between 3320 and 3380 G. This approach is denoted as "intensity".
2. We baseline-corrected the spectra to remove only the very broad contribution (see Figure S9). To this end, we fitted a 5th degree polynomial baseline to the data at 2600–3000 G and 3430–4000 G and subtracted it from the experimental data. Subsequently, to obtain the signal amplitude as a measure for radical amounts, we subtracted the minimum from the maximum baseline-corrected intensity in a magnetic field window between 3320 and 3380 G. This approach is denoted as "intensity (broad baseline)".
3. We baseline-corrected the spectra in a narrower region to remove both the very broad (see Figure S8) and the less broad (see Figure S9) background contributions. To this end, we fitted a 5th degree polynomial baseline to the data at 3230–3280 G and 3430–3500 G and subtracted it from the experimental data. Subsequently, to obtain the signal amplitude as a measure for radical amounts, we subtracted the minimum from the maximum baseline-corrected intensity in a magnetic field window between 3320 and 3380 G. This approach is denoted as "intensity (narrow baseline)".
4. We first integrated the spectra to obtain microwave absorption signals. These were baseline-corrected by fitting a 5th degree polynomial baseline and subsequently integrated again to obtain the double integral of the derivative spectrum as a measure for radical amounts. The boundaries of the baseline and second integration were chosen based on the broadness of signals. For spectra recorded after 60 min of milling, the baseline was fitted to data at 3230–3325 G and 3385–3500 G, and the second integration was performed between 3325 and 3385 G. For spectra recorded after 120 min of milling, the baseline was fit to the data at 3230–3295 G and 3410–3500 G, and the second integration was performed between 3295 and 3410 G. For spectra recorded after 240 and 480 min of milling, the baseline was fitted to data at 3230–3280 G and 3430–3500 G, and the second integration was performed between 3280 and 3430 G. This approach is denoted as "integrated".

5. We first baseline-corrected the data to remove both the very broad and the less broad background contributions as described in the "intensity (narrow baseline)" procedure. Subsequently, we used the Python EPRsim package to simulate the resulting spectra in the regions between 3300 and 3400 G. To this end, we manually assigned an appropriate *g* value between 1.998 and 2.001, and optimized the simulation in terms of linewidth (Gaussian and Lorentzian) and signal height. Subsequently, we integrated the simulation two-fold between 3250 and 3450 G to obtain a measure for radical amounts. This approach is denoted as "simulation".

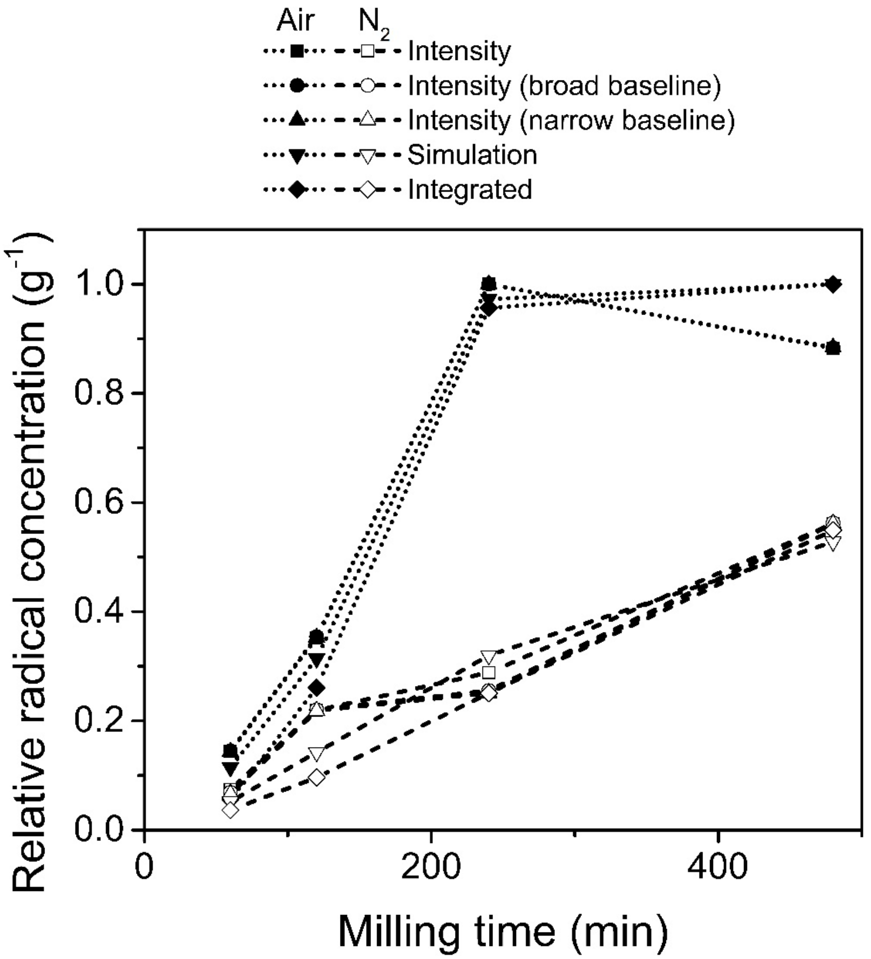

Figure S11. Relative radical concentrations versus time when milling under air or N_2 according to different methods: Signal amplitude (intensity), two-fold integrated signal, signal amplitude after broad and narrow baseline correction, and fitting of a simulated spectrum to experimental data and two-fold integration.

S.F. Particle Size Data

Optical microscopy images were taken using the imaging module on a Renishaw inVia Raman Microscope. Images take were of unmilled PS feedstock and residues from depolymerization of 1 g of PS inside a 25 mL stainless steel reactor with eight 10 mm stainless steel balls in air or N_2 atmospheres, milled at 30 Hz.

Unmilled PS feedstock.

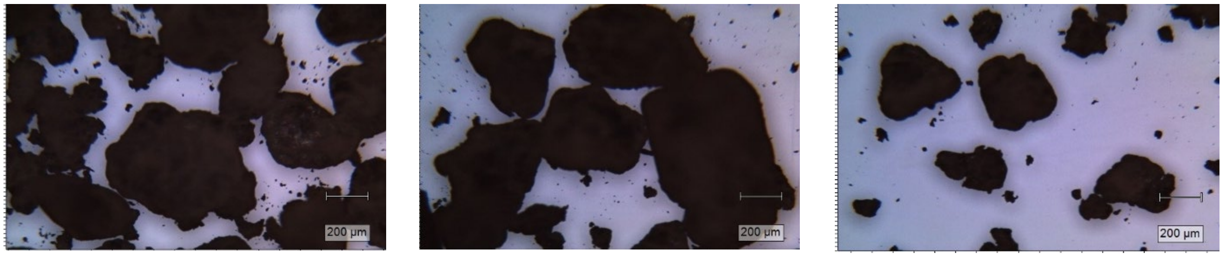

Air atmosphere; rows from top to bottom: residue at 60, 120, 240 and 480 min of ball milling.

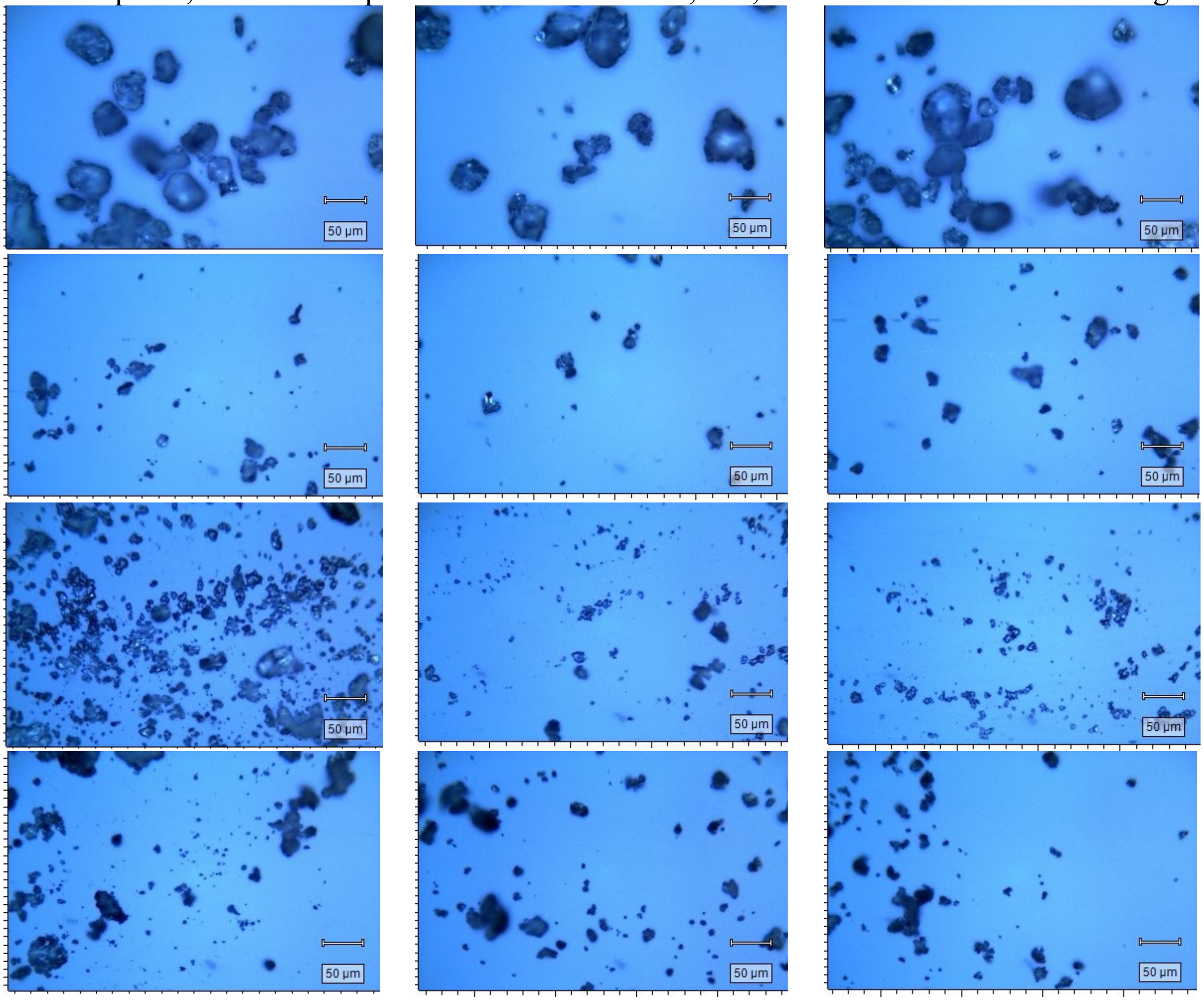

Figure S12. Microscopy images of unmilled PS feed and PS residues milled for select instants in time. Each row contains three images of residue sampled from the same experiment at one time under air or N_2 gas atmosphere.

N_2 atmosphere; rows from top to bottom: residue at 60, 120, 240 and 480 min.

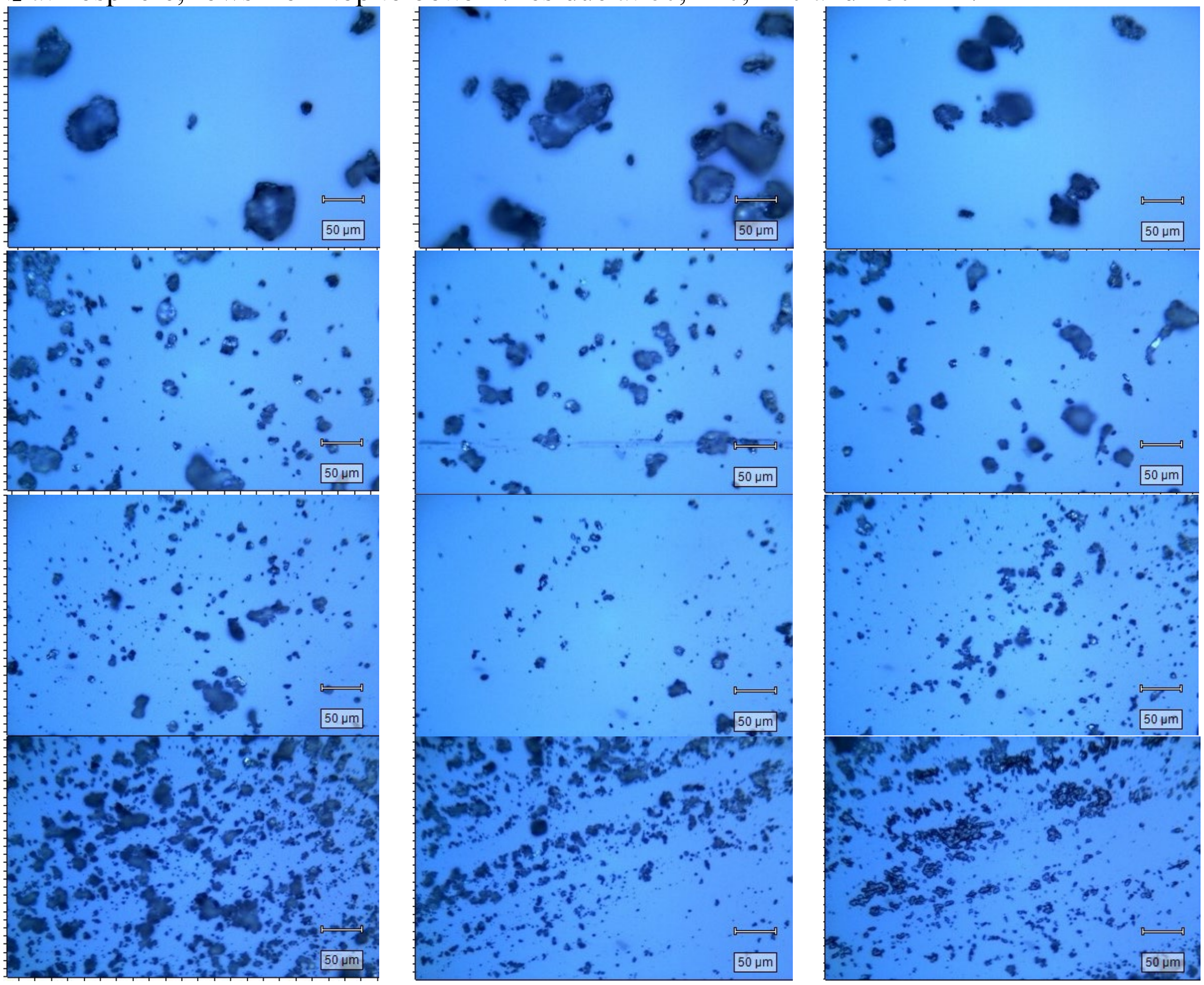

Figure S12 cont'd.

The images depicted in Figure S12 were used to measure a selection of particle sizes based on the scale bar (50 µm) present in each image. Particles were sampled from all three images taken for each atmosphere and milling time. A single particle size was determined by measuring the particle dimensions along two perpendicular axes and taking the geometric mean of the two measurements. Resultant histograms depicting the particle size distribution obtained by sampling from images are provided as Figure S13. The average size computed from these distributions is plotted with milling time in Figure S14.

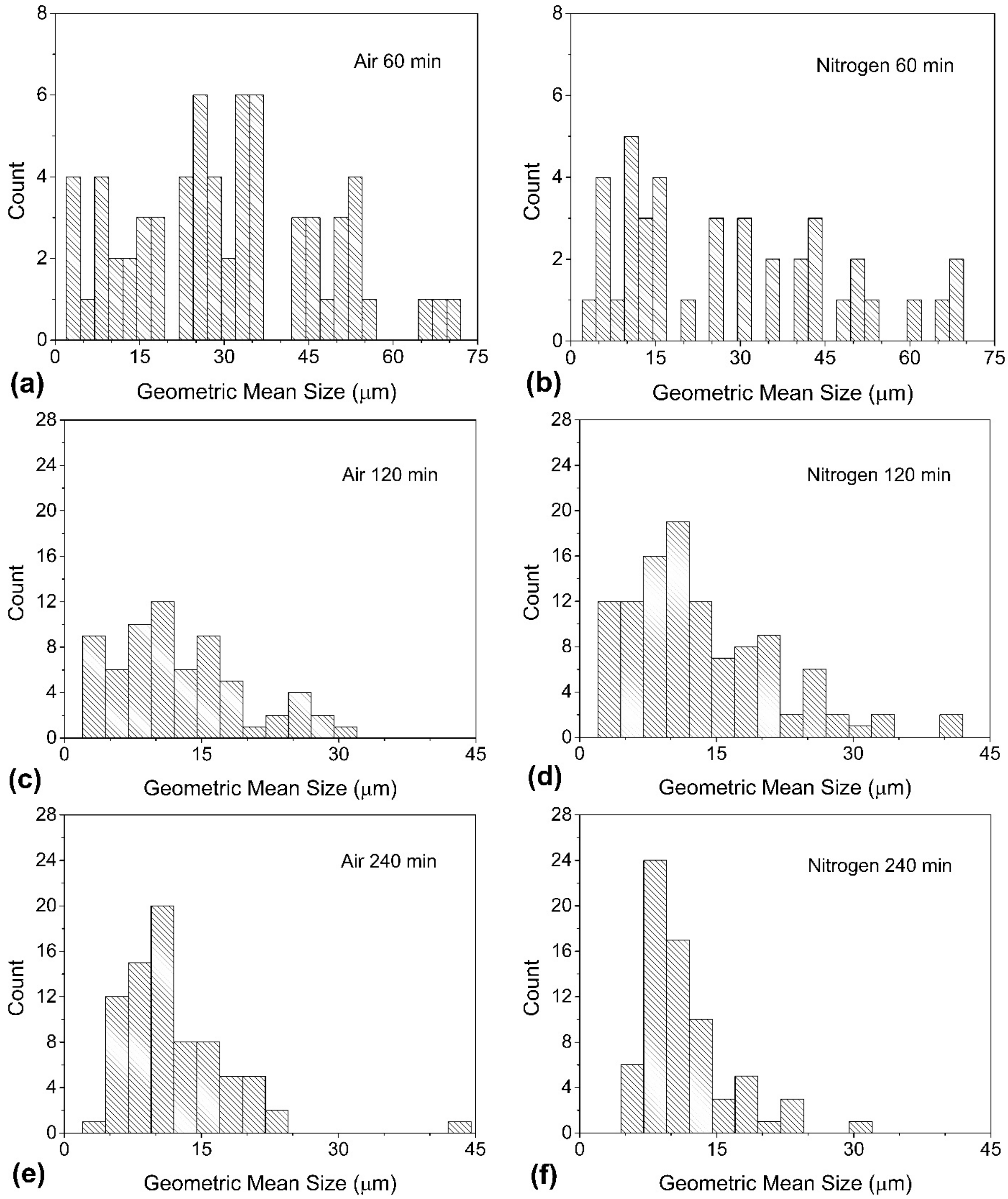

Figure S13. Histograms of particle sizes (geometric mean size) at sampled milling times and atmospheres obtained from microscopy images in Figure S12. The bin width increments by 2.5 μm for all plots except subfigure S13i, which is binned at 5 μm increments due to the large spread of particle sizes.

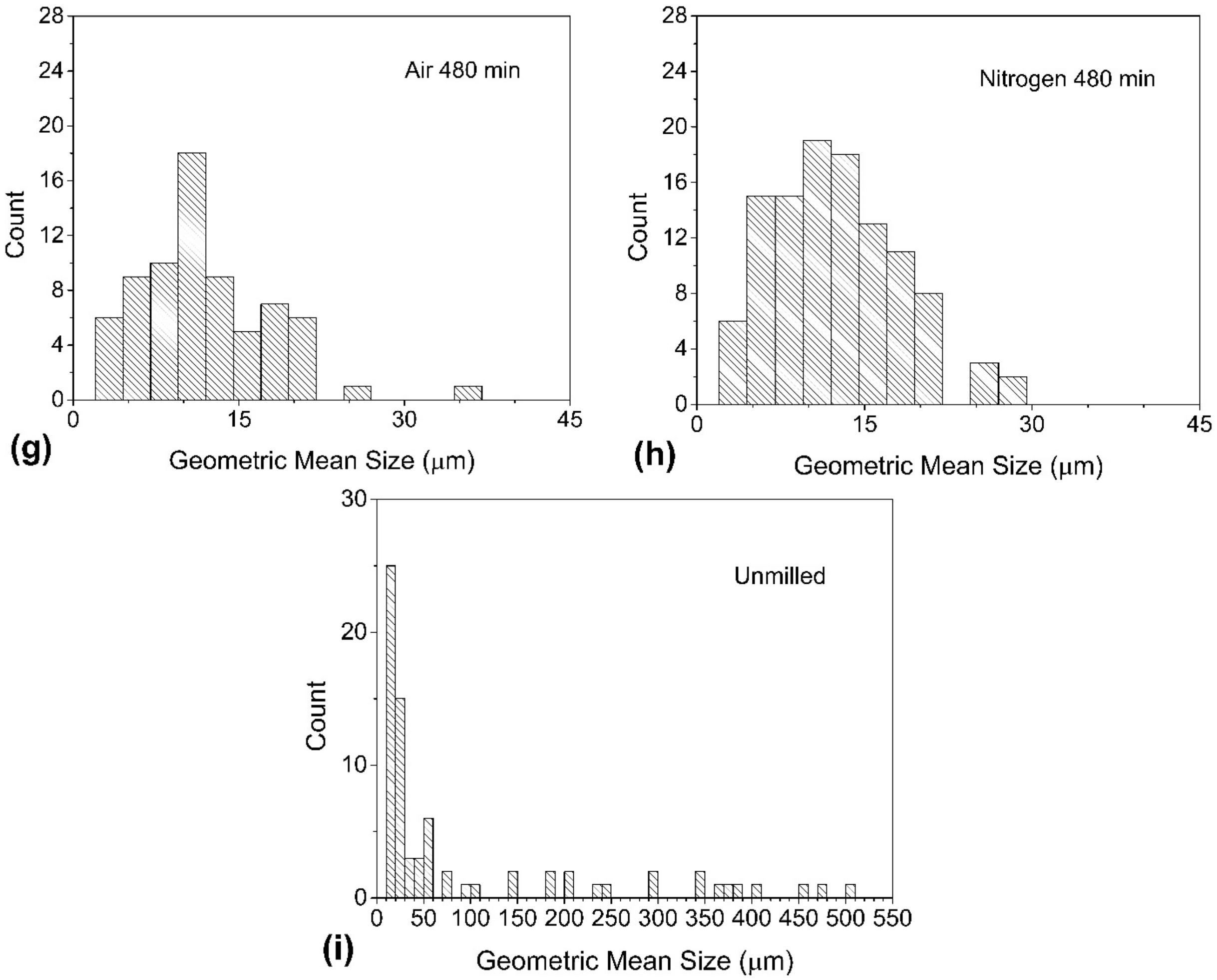

Figure S13 cont'd.

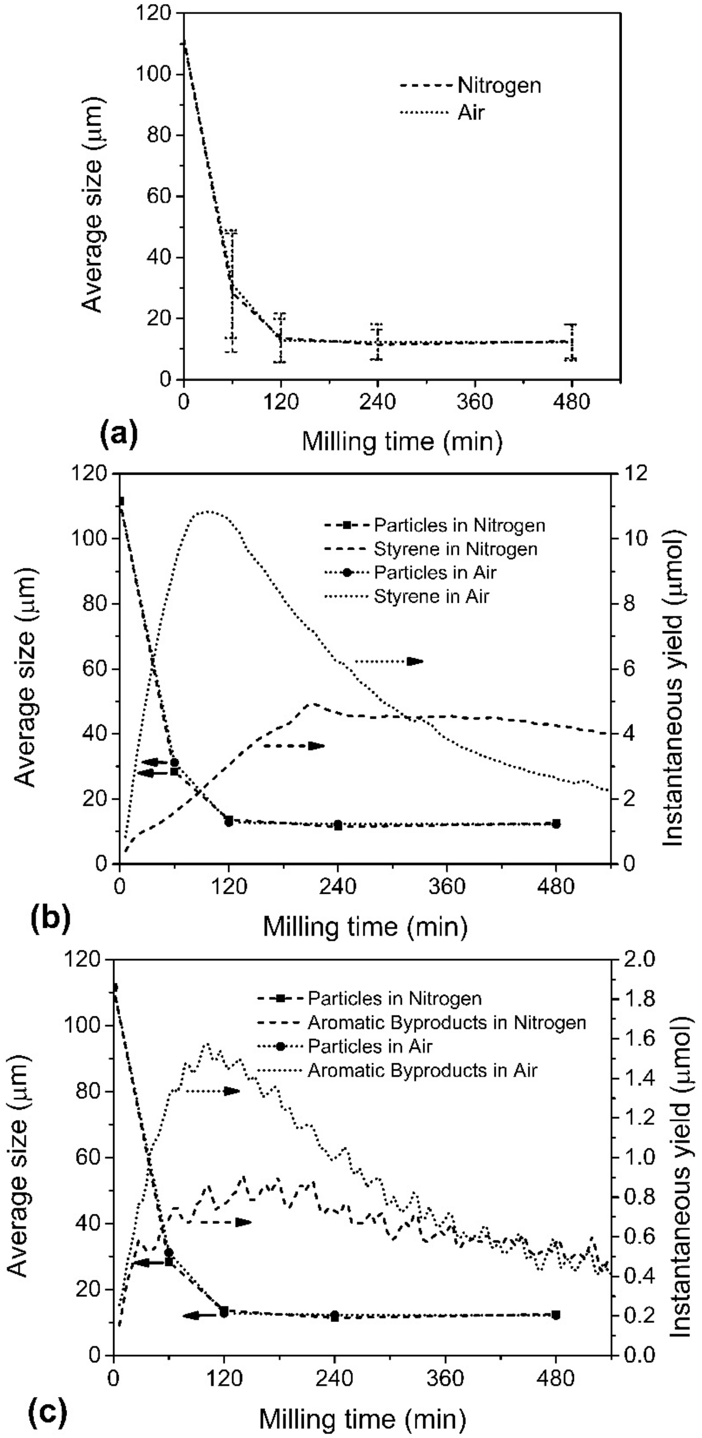

Figure S14. Average particle size versus milling time computed from histograms in Figure S13 (a) and the same data plotted alongside instantaneous styrene (b) and aromatic byproduct (c) yields.

S.G. Miscellaneous Images

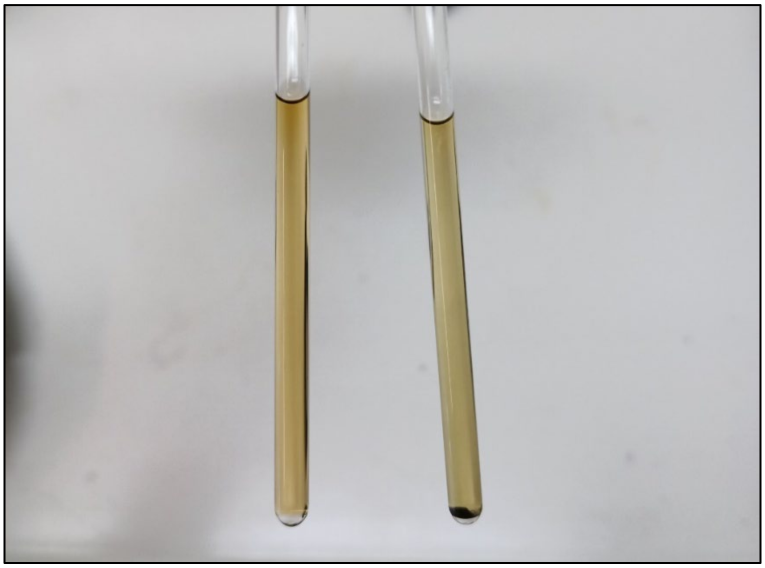

Figure S15. Optical photograph of filtered $CDCl_3$ solutions of PS residues milled for 12 hours under N_2 (left) and air atmospheres showing a distinctly yellowish tint. Residues are also soluble in tetrahydrofuran.

References

(1) Chang, Y.; Blanton, S. J.; Andraos, R.; Nguyen, V. S.; Liotta, C. L.; Schork, F. J.; Sievers, C. Kinetic Phenomena in Mechanochemical Depolymerization of Poly(Styrene). *ACS Sustain. Chem. Eng.* **2024**, *12* (1), 178–191. https://doi.org/10.1021/acssuschemeng.3c05296.

(2) Bossoni, L.; Labra-Muñoz, J. A.; van der Zant, H. S. J.; Čaluković, V.; Lefering, A.; Egli, R.; Huber, M. In-Depth Magnetometry and EPR Analysis of the Spin Structure of Human-Liver Ferritin: From DC to 9 GHz. *Phys. Chem. Chem. Phys.* **2023**, *25* (40), 27694–27717. https://doi.org/10.1039/D3CP01358H.
